# Curcumin Nanodelivery for Tumor-Associated Macrophage Reprogramming and Immune-Checkpoint Sensitization: Engineering the Tumor Immune Microenvironment

**DOI:** 10.3390/pharmaceutics18101295

**Published:** 2026-10-13

**Authors:** Juie Nahushkumar Rana, Sohail Mumtaz

**Affiliations:** 1 Fels Cancer Institute for Personalized Medicine, Lewis Katz School of Medicine, Temple University, Philadelphia, PA 19140, USA; 2 Department of Chemical and Biological Engineering, Gachon University, 1342 Seongnamdaero, Sujeong-gu, Seongnam-si 13120, Republic of Korea

**Keywords:** curcumin, nanodelivery, tumor-associated macrophages, macrophage reprogramming, tumor immune microenvironment, STAT3, STAT6, PD-1, PD-L1, immune-checkpoint blockade

## Abstract

**Background/Objectives:** Curcumin possesses antineoplastic and immunomodulatory activities. However, poor aqueous solubility, chemical instability, rapid conjugative metabolism, and limited systemic exposure restrict meaningful concentrations within tumors. Numerous reviews have already addressed curcumin nanoformulations, improved bioavailability, and general anticancer effects. However, delivery architecture, cell-specific curcumin exposure, TAM functional reprogramming, macrophage–lymphocyte crosstalk, and checkpoint response remain insufficiently integrated. This review connects these components while distinguishing experimentally demonstrated links from mechanistic inference and examines whether engineered curcumin nanodelivery can remodel TAM-associated biology to improve responsiveness to PD-1/PD-L1-directed therapy. **Methods:** A structured narrative review was conducted using experimental studies of curcumin delivery, macrophage targeting and reprogramming, intracellular signaling, macrophage–lymphocyte crosstalk, and checkpoint regulation. **Results:** Evidence supports curcumin-associated modulation of STAT3/STAT6, PI3K–AKT, context-dependent NF-κB, HIF-1α, redox, and metabolic pathways, accompanied by reduced immunosuppressive mediators and enhanced antitumor macrophage functions. Emerging nanoplatforms further report TAM remodeling, immunogenic cell death, enhanced T-cell responses, and additional therapeutic benefit in checkpoint-containing regimens. However, these endpoints are usually distributed across different experimental systems rather than one complete causal sequence. We therefore distinguish validated TAM targeting from tumor accumulation, biomimetic tumor homing, secondary TAM exposure, and combination benefit from demonstrated checkpoint sensitization. **Conclusions:** We propose a multidimensional D1–D8 evidence framework evaluating pharmaceutical performance, tumor exposure, TAM-specific uptake and intracellular parent curcumin, pathway modulation, functional TAM reprogramming, downstream immune restoration, checkpoint-therapy response, and translational relevance as independent domains. This prevents efficacy in one domain from being interpreted as proof of unmeasured links elsewhere and advances cell-resolved, mechanism-driven curcumin immunonanomedicine.

## 1. Introduction

Curcumin (Cur) is a pleiotropic diarylheptanoid with experimentally documented effects on inflammatory transcription, survival signaling, cell death, redox control, and immune regulation. Its pharmacological attractiveness is matched by a long-standing formulation problem: native curcumin is poorly soluble in water, undergoes chemical degradation and extensive intestinal/hepatic conjugation, and produces low circulating concentrations after conventional oral dosing [1,2,3,4,5,6,7]. Those limitations are especially relevant to immunomodulation. A concentration that suppresses signaling in a tumor cell monolayer does not establish that free curcumin, given systemically, reaches the same intracellular exposure in stromal or immune compartments in vivo. This exposure gap is one reason why the field has shifted from simply describing curcumin’s anticancer effects toward engineering carriers that alter solubility, stability, biodistribution, cellular uptake, and release [1,2,6,7].

Curcumin can modulate NF-κB-dependent transcription and other signaling programs that intersect with inflammation, myeloid-cell function, and antitumor immunity [8,9,10]. Recent reviews have catalogued curcumin nanoplatforms, immune effects, or combinations with immunotherapy [11,12,13,14,15]. Yet these studies are often organized in parallel: one review describes liposomes, micelles, polymeric nanoparticles, and biomimetic carriers; another describes macrophages, lymphocytes, cytokines, or immune checkpoints. The mechanistic bridge between the two is less developed. For a delivery system to matter immunologically, its architecture must change where and when curcumin is available, which cells actually receive it, which intracellular pathways are engaged, and whether those changes propagate through the tumor immune ecosystem.

Tumor-associated macrophages (TAMs) are a particularly useful compartment in which to test that bridge. They can comprise a substantial fraction of the immune infiltrate, are linked to angiogenesis, invasion, metastatic dissemination, therapy resistance, and T-cell suppression, and can be pharmacologically depleted, redirected, or reprogrammed [16,17,18,19,20,21,22,23]. However, a binary M1-versus-M2 model is inadequate for human cancer. Consensus nomenclature and transcriptomic studies show a continuum of activation states, while single-cell atlases reveal tumor-, tissue-, and patient-specific macrophage programs that cannot be reduced to one CD86/CD206 axis [16,17,24,25,26,27]. Accordingly, this review uses M1-like and M2-like terms only as operational shorthand when they match the design of the cited experiment. The more important endpoints are cell identity, transcriptional state, cytokine output, phagocytic behavior, antigen-presentation capacity, metabolic program, spatial localization, and functional influence on other immune cells.

TAMs also sit at a mechanistic intersection with immune-checkpoint therapy. Tumor-derived chemokines recruit monocytes and retain macrophages; CSF1/CSF1R signaling supports macrophage survival; IL-10, TGF-β, hypoxia, lactate, and PI3Kγ-dependent programs can reinforce suppressive states; and macrophages can physically or functionally restrict cytotoxic T-cell access [28,29,30,31,32,33,34,35,36,37]. TAMs themselves may express PD-1, while myeloid cells can sequester therapeutic anti-PD-1 antibodies or participate in adaptive resistance pathways [37,38,39]. These observations make macrophage reprogramming a plausible sensitization strategy rather than merely an anti-inflammatory adjunct. The critical question is therefore not whether curcumin is broadly ‘immunomodulatory’, but whether delivery-controlled curcumin exposure can disrupt macrophage-centered resistance circuits strongly enough to improve checkpoint responsiveness.

Direct experimental literature at this intersection is now emerging. M2pep-functionalized chitosan-curcumin nanoparticles have been reported to promote macrophage uptake and TAM reprogramming in triple-negative breast cancer, with greater antitumor activity observed when the nanoparticles were combined with anti-PD-L1 than with nanoparticle treatment alone [40]. However, because the in vivo comparison lacked an anti-PD-L1-monotherapy arm, these findings support a checkpoint-combination benefit but do not independently establish checkpoint sensitization or synergy. Other curcumin nanoplatforms combine tumor targeting, pH-responsive release, photothermal or photodynamic components, checkpoint-blocking ligands, or immunogenic cell death (ICD) programs [41,42,43,44,45,46,47,48,49]. Earlier dendrosomal, phytosomal, and liposomal studies also provide evidence that formulation can influence macrophage/microglial polarization and antitumor immune activity [50,51,52,53]. At the same time, some frequently cited historical evidence requires caution: the 2013 hydrazinocurcumin nanoparticle study reported STAT3-associated TAM re-education and antitumor activity [54], but a subsequent Expression of Concern identified unresolved concerns regarding control and image data [55]. We therefore retain this study only as qualified historical evidence rather than as a secure mechanistic pillar.

**Figure 1:**
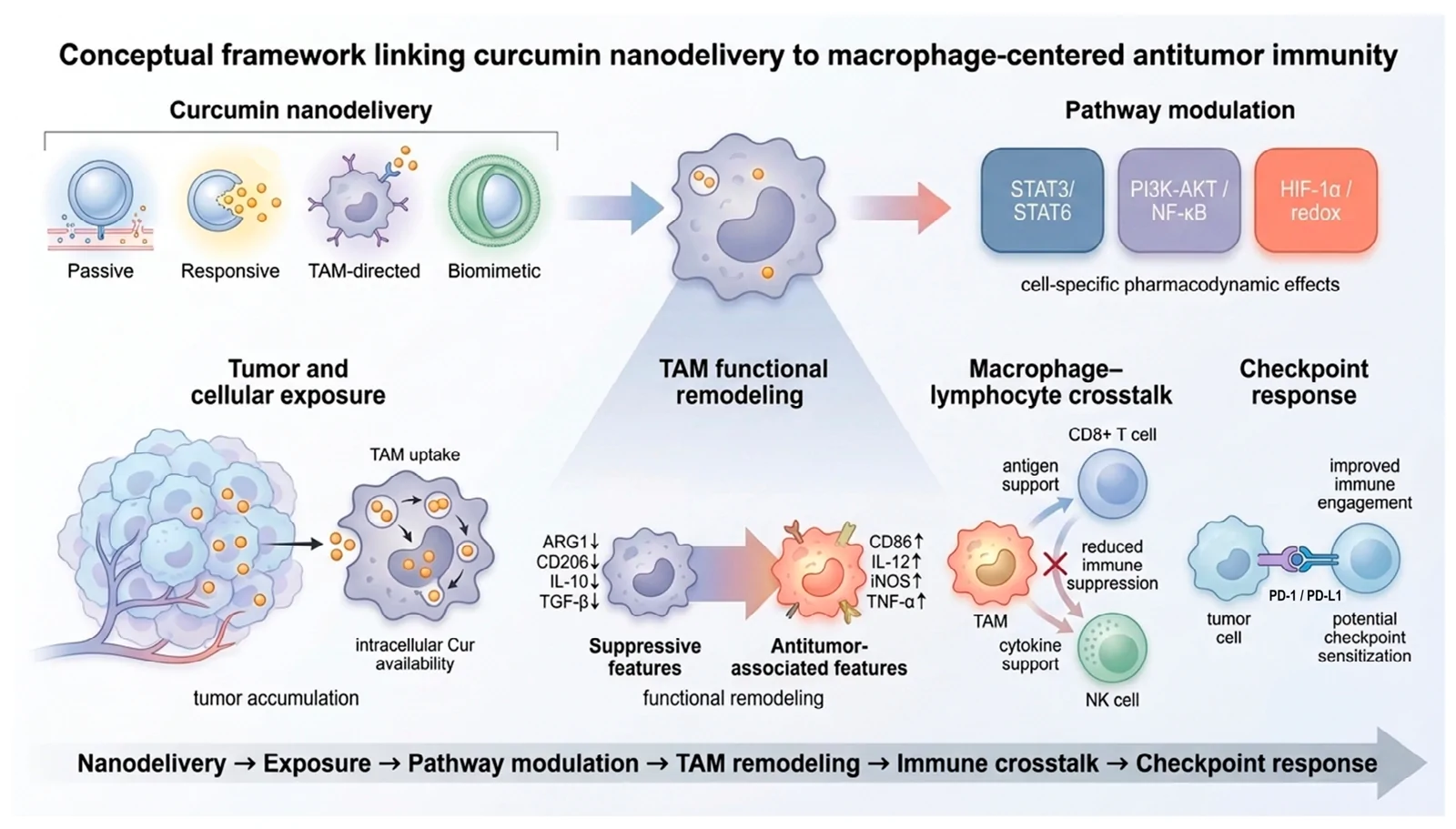
Conceptual framework linking curcumin nanodelivery to macrophage-centered antitumor immunity. Curcumin nanodelivery platforms can influence tumor accumulation, tumor-associated macrophage (TAM) uptake, and intracellular Cur availability. These exposure changes may alter cell-specific pharmacodynamic pathways, including STAT3/STAT6, PI3K-AKT/NF-κB, and HIF-1α/redox networks. Downstream effects can include reduced suppressive TAM features and increased antitumor-associated functions, with consequences for macrophage–lymphocyte crosstalk, CD8^+^ T-cell and NK-cell support, and PD-1/PD-L1-directed immune responses. The figure summarizes the mechanistic sequence evaluated in this review; it does not imply that all steps have been demonstrated within a single experimental system.

This review therefore focuses on a defined mechanistic axis: delivery architecture → spatial and intracellular curcumin exposure → cell-specific pharmacodynamic pathway modulation → causal pathway attribution where supported by perturbation evidence → functional TAM reprogramming → macrophage–lymphocyte crosstalk → checkpoint response. The novelty is not a claim that curcumin, TAMs, or PD-1/PD-L1 have never been reviewed. Rather, it is the explicit integration of these components through a multidimensional evidence framework in which formulation performance, tumor exposure, TAM-specific uptake and intracellular drug exposure, pathway modulation, TAM function, downstream immunity, checkpoint response, and translational relevance are evaluated independently rather than inferred from one another. This framework also clarifies where direct evidence remains limited: where direct curcumin-TAM-checkpoint evidence is limited, established TAM nanomedicine and macrophage signaling studies are used as mechanistically supportive evidence, and the distinction is stated rather than blurred. Figure 1 summarizes the proposed conceptual sequence linking Cur nanodelivery and cellular exposure to pharmacodynamic pathway modulation, TAM functional remodeling, macrophage–lymphocyte crosstalk, and potential checkpoint response.

### 1.1. Literature Identification Approach

This structured narrative review searched PubMed/MEDLINE and Google Scholar, supplemented by citation chaining, publisher-indexed records, and targeted searches of studies available up to 18 September 2026. Representative combinations included (“curcumin” OR “curcuminoid”) AND (“tumor-associated macrophage” OR “macrophage reprogramming”) AND (“nanoparticle” OR “nanodelivery”), and (“curcumin”) AND (“PD-1” OR “PD-L1” OR “checkpoint blockade”). Additional terms included M2pep, CD206, CSF1R, STAT3, STAT6, NF-κB, PI3K/AKT, HIF-1α, tumor immune microenvironment, and immunogenic cell death. Peer-reviewed experimental studies were prioritized when they reported Cur delivery, macrophage uptake or function, pathway modulation, immune effects, PD-1/PD-L1 regulation, or checkpoint-combination efficacy. Reviews were used for field mapping and citation discovery [11,12,13,14,15]. Studies limited to general Cur cytotoxicity, formulation performance without relevant immune data, or unsupported pathway inference were excluded from the core evidence set. Records were screened first for relevance to Cur nanodelivery, macrophage/TAM biology, immune modulation, or checkpoint-related outcomes, after which potentially eligible experimental studies were examined in full text and citation-chained to identify additional primary reports.

Data from the principal experimental studies were extracted for formulation characteristics, model, macrophage source, exposure evidence, pathway and immune readouts, checkpoint design, comparator groups, and study limitations. Evidence assignments were checked against the primary reports, and ambiguous findings were classified conservatively. The principal studies assessed are listed in Supplementary Table S1. The chemical identity and evidentiary classification of curcumin-related compounds and formulations are summarized in Supplementary Table S2. Parent Cur was distinguished from chemically distinct derivatives and multi-agent formulations. Hydrazinocurcumin and tetrahydrocurcumin were treated separately, while TriCurin was considered a multi-agent formulation containing Cur, epicatechin gallate, and resveratrol [53,54,56]. These chemically distinct systems were used only as qualified supportive evidence and were not treated as direct evidence for parent curcumin. The hydrazinocurcumin study was also interpreted in light of its subsequent Expression of Concern [55]. Because this was a structured narrative rather than a formal systematic review, a PRISMA flow diagram was not generated.

### 1.2. Methods: Use of Generative Artificial Intelligence

**Figure 2:**
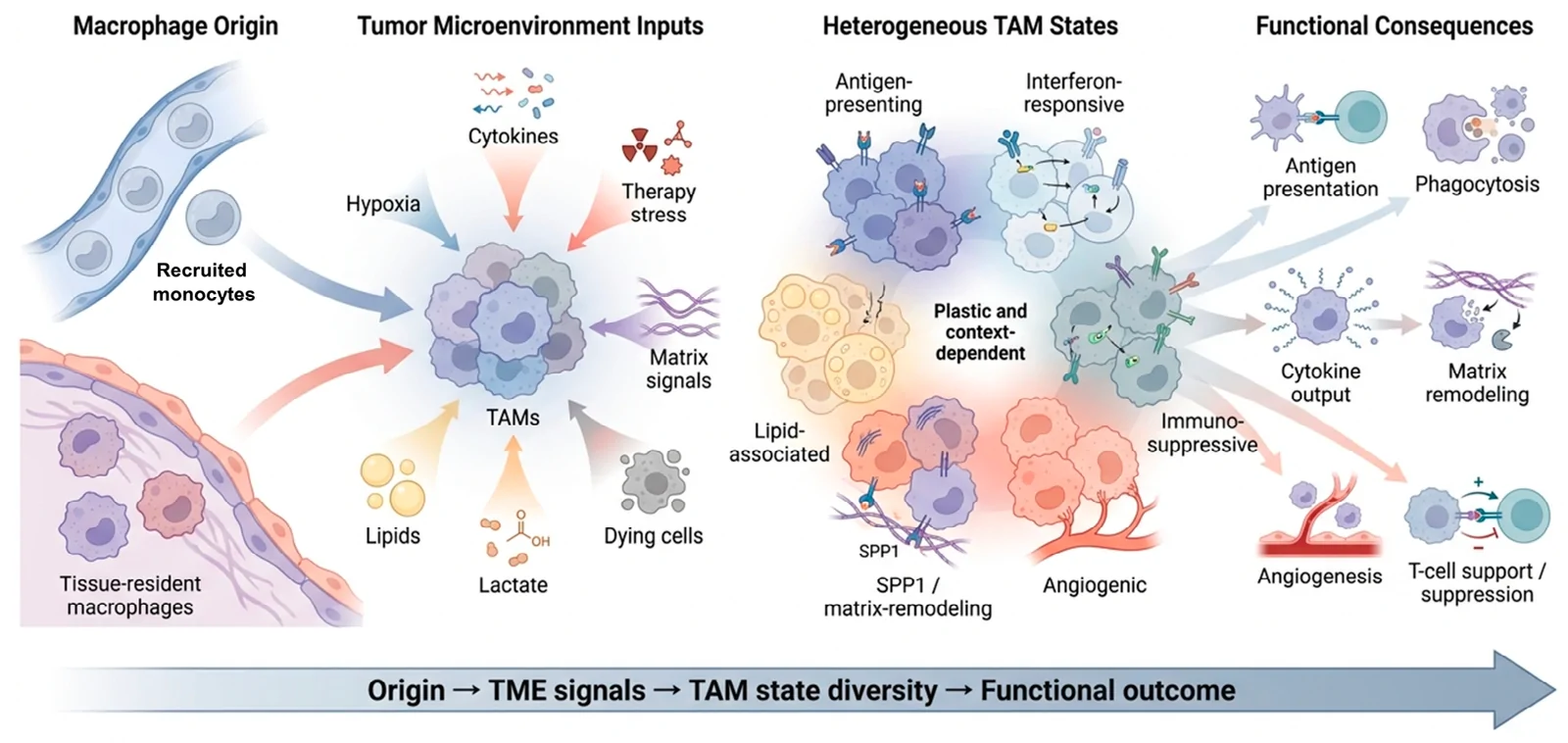
Heterogeneous tumor-associated macrophage states relevant to curcumin immunonanomedicine. Tumor-associated macrophages (TAMs) arise from recruited monocytes and tissue-resident macrophage populations and are continuously shaped by tumor-microenvironmental signals, including cytokines, hypoxia, lactate, lipids, matrix-derived cues, dying cells, and therapy-induced stress. These inputs generate overlapping and plastic macrophage states associated with antigen presentation, interferon responses, lipid handling, SPP1-linked matrix remodeling, angiogenesis, and immune suppression. The resulting functional outputs include phagocytosis, cytokine production, matrix remodeling, vascular regulation, and support or suppression of T-cell activity. The figure emphasizes that TAM states are dynamic and context-dependent rather than fixed M1/M2 categories.

**Figure 3:**
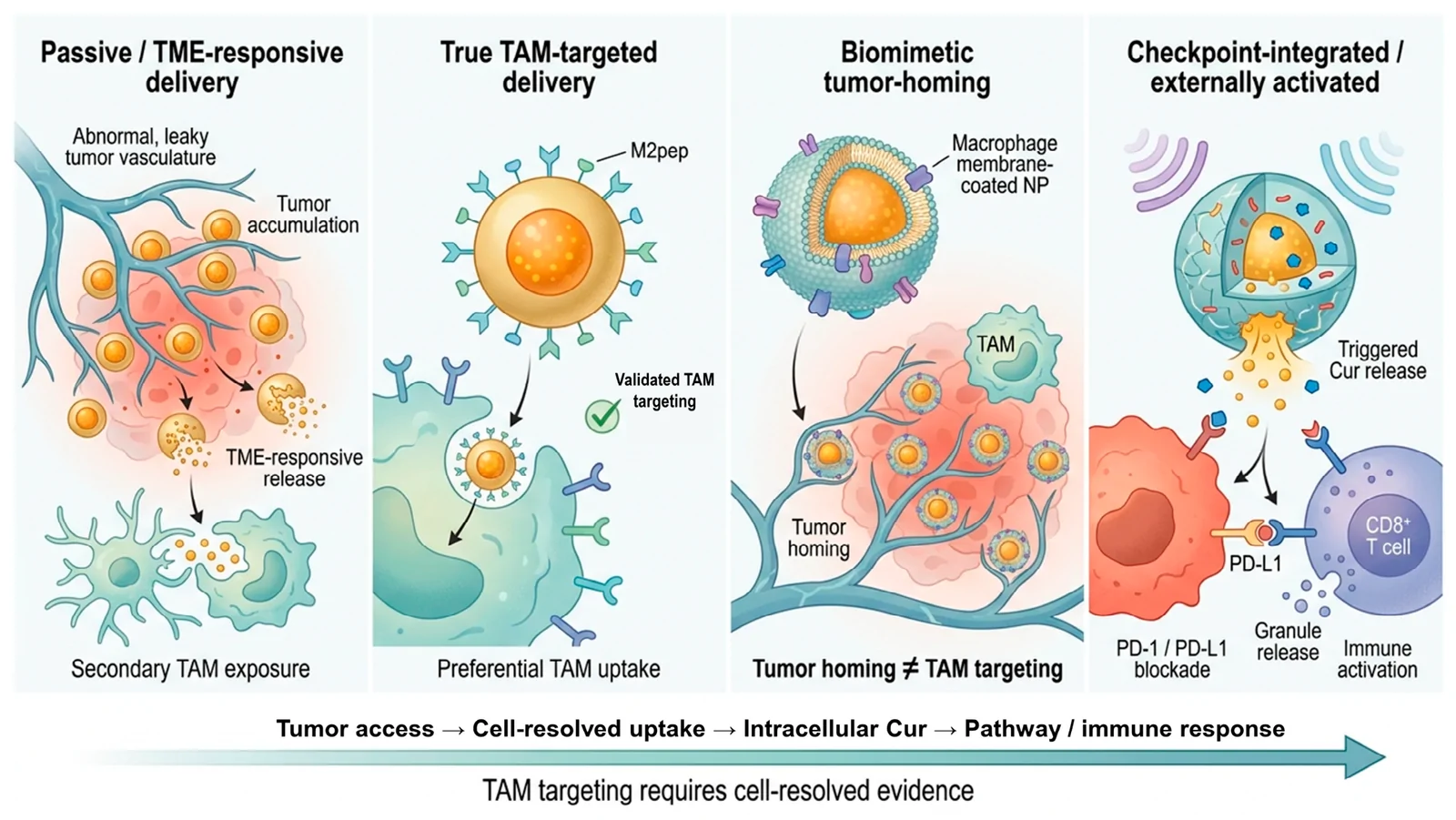
Engineering classes of curcumin immunonanomedicine and the distinction between TAM targeting and secondary TAM exposure. Passive or tumor-microenvironment-responsive systems can increase tumor accumulation and local Cur release, but subsequent TAM exposure does not establish macrophage-specific targeting. True TAM-directed delivery requires validated targeting and preferential macrophage uptake. Biomimetic systems can enhance tumor homing without necessarily targeting TAMs, whereas checkpoint-integrated or externally activated platforms combine Cur release with immune-checkpoint or stimulus-responsive mechanisms. The figure emphasizes that tumor access, cell-resolved uptake, intracellular Cur availability, and downstream pathway or immune responses are distinct evidentiary steps, and that claims of TAM targeting require cell-resolved validation.

**Figure 4:**
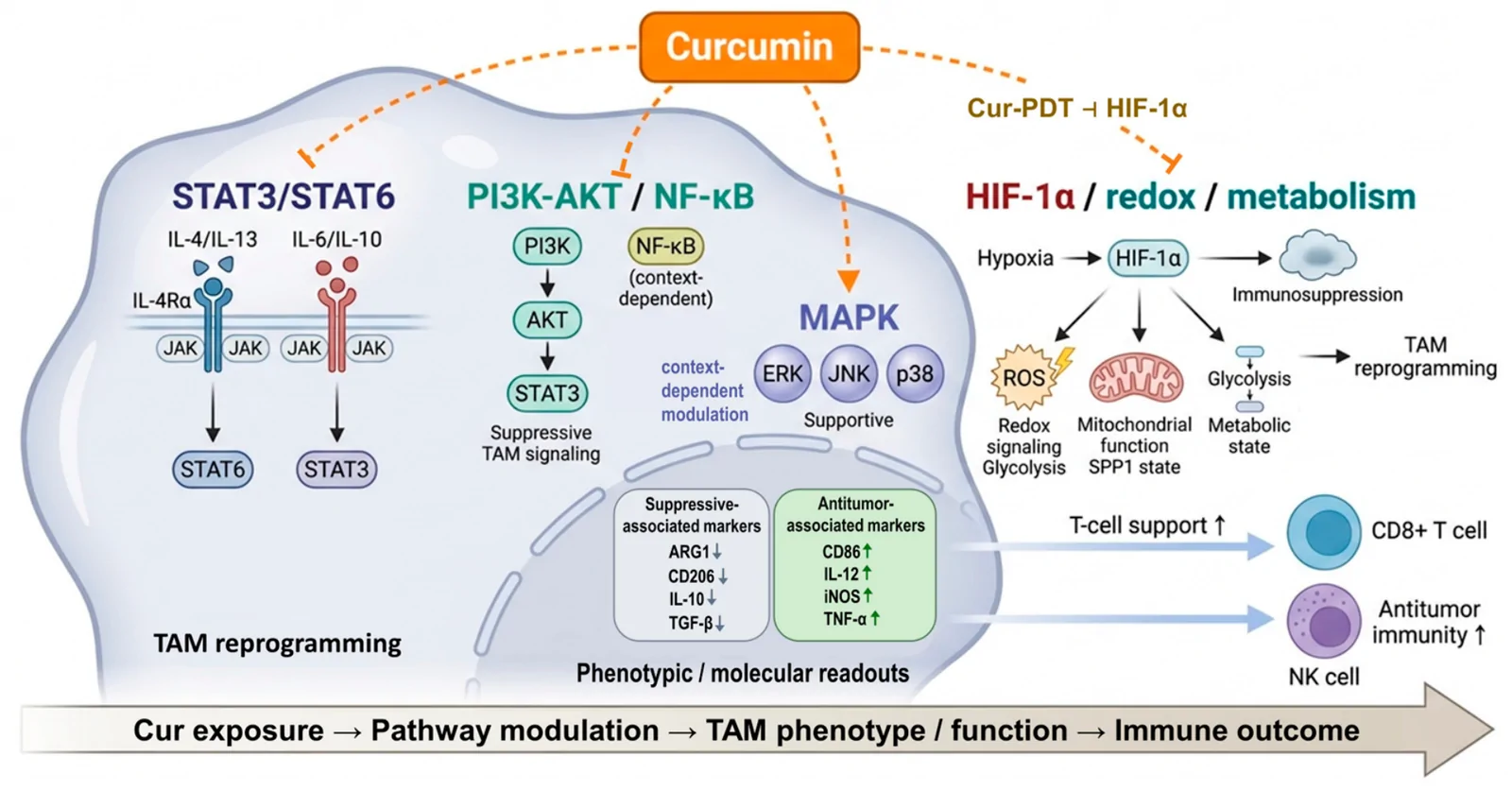
Molecular pathway map of curcumin-associated tumor-associated macrophage (TAM) reprogramming. Curcumin-associated modulation of STAT3/STAT6, PI3K–AKT/STAT3, NF-κB, MAPK, and HIF-1α/redox/metabolic pathways is shown with inhibitory, activating, or context-dependent relationships distinguished explicitly. Changes in ARG1, CD206, IL-10, TGF-β, CD86, IL-12, iNOS, and TNF-α are presented as phenotypic/molecular readouts rather than proof of macrophage function. Functional consequences and downstream immune outcomes are indicated only where independently measured.

**Figure 5:**
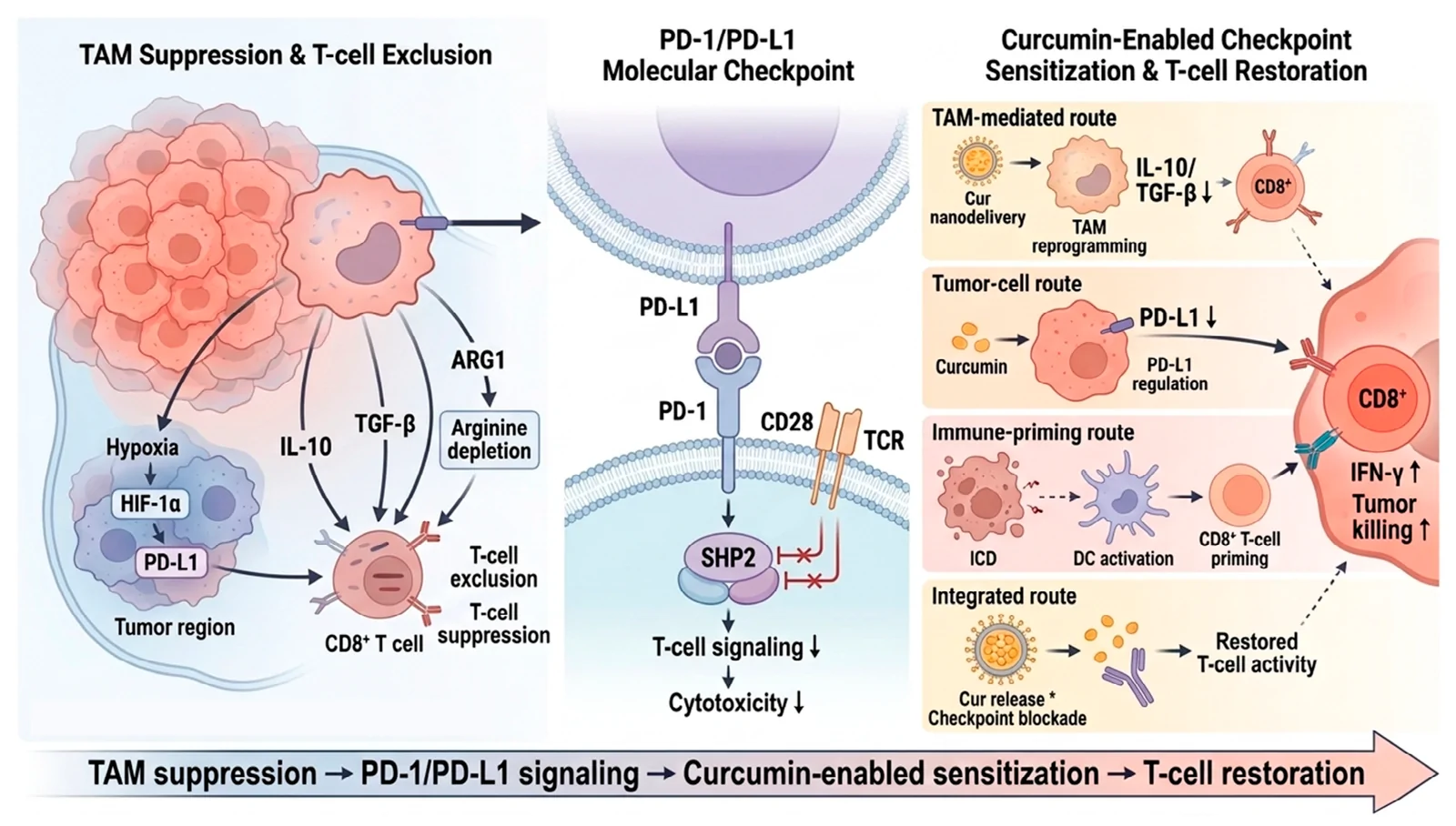
TAM–T-cell–PD-1/PD-L1 molecular axis and routes of curcumin-enabled checkpoint sensitization. Immunosuppressive TAMs contribute to T-cell exclusion and dysfunction through IL-10, TGF-β, ARG1-dependent arginine depletion, hypoxia-associated HIF-1α signaling, and PD-L1 expression. Engagement of PD-L1 with PD-1 on CD8^+^ T cells promotes SHP2 recruitment and attenuation of CD28/TCR signaling, thereby reducing T-cell activation and cytotoxicity. Curcumin nanodelivery may enhance checkpoint responsiveness through several mechanistically distinct routes: TAM reprogramming with reduced IL-10/TGF-β output, modulation of tumor-cell PD-L1 expression, induction of ICD with dendritic-cell activation and CD8^+^ T-cell priming, and checkpoint-integrated nanocarriers combining curcumin release with PD-1/PD-L1 blockade. These routes may converge on restoration of cytotoxic T-cell function, increased IFN-γ production, and enhanced tumor-cell killing. Solid arrows denote established or experimentally measured relationships, whereas dashed arrows indicate supportive or mechanistically inferred links across cellular compartments.

**Figure 6:**
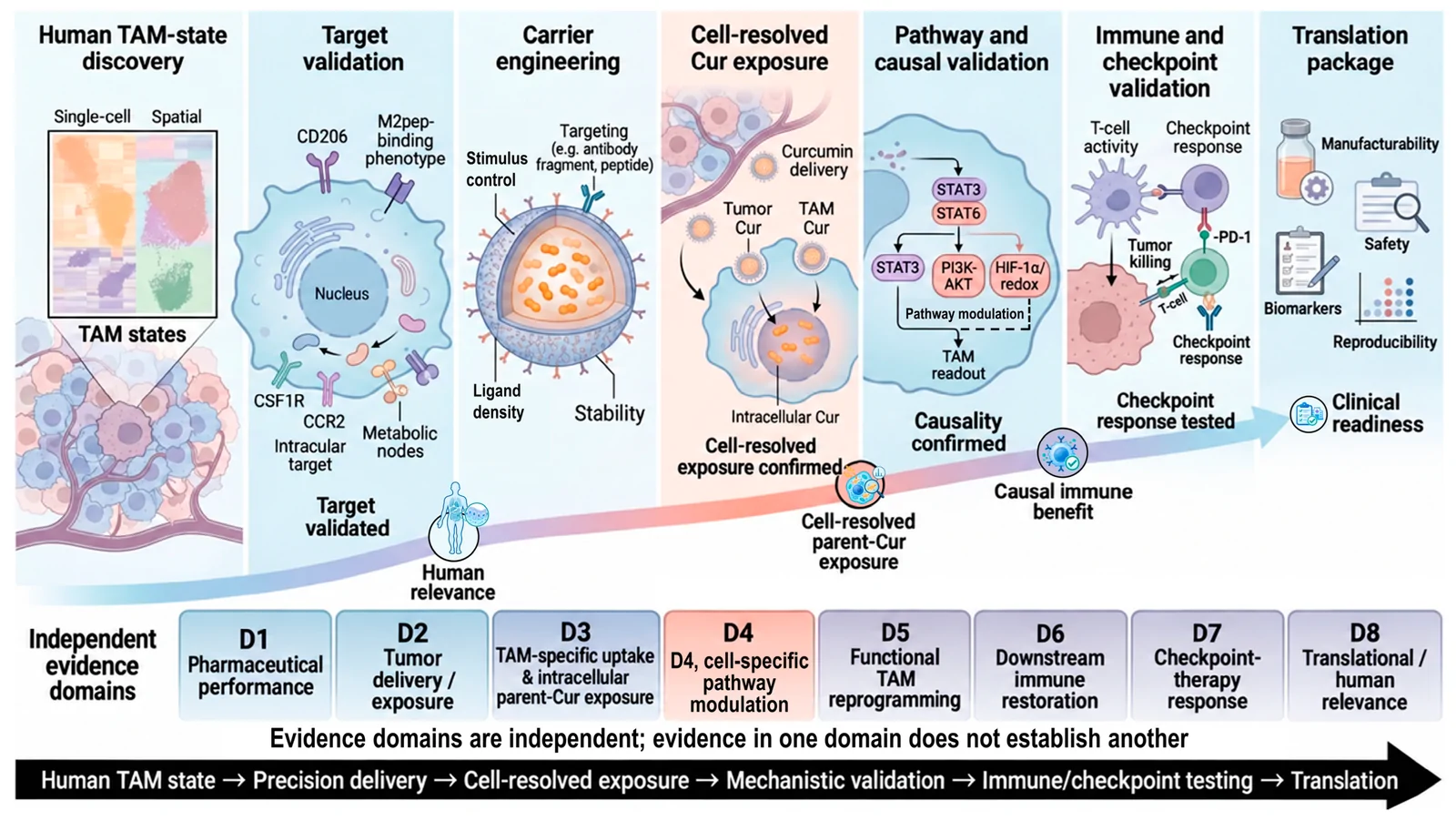
Evidence-to-translation framework for curcumin immunonanomedicine. The upper workflow outlines a prospective translational strategy progressing from human TAM-state discovery and target validation to carrier engineering, cell-resolved Cur exposure, pathway and causal validation, immune/checkpoint testing, and clinical readiness. The lower panel presents eight independent evidence domains: D1, pharmaceutical performance; D2, tumor delivery/exposure; D3, TAM-specific uptake and intracellular parent curcumin exposure; D4, cell-specific pathway modulation; D5, functional TAM reprogramming; D6, downstream immune restoration; D7, checkpoint-therapy response; and D8, translational/human relevance. In D1–D8, “D” denotes “domain” and is distinct from the study-level evidence code D, which denotes directly demonstrated evidence. The domains are not cumulative, and evidence in one domain does not establish another. Within D4, directly measured pathway modulation does not by itself establish causal pathway dependence; causal attribution requires perturbation/rescue evidence, while direct molecular target engagement requires independent drug–target binding or occupancy evidence.

During the preparation of this work, all literature searches, critical evaluation of studies, data synthesis, interpretation of findings, and initial drafting of the manuscript were performed manually by the authors. The fundamental concepts and scientific conclusions presented in this review were derived solely from the authors’ independent analysis of the published literature. ChatGPT (OpenAI, version GPT-5.6 Sol, San Francisco, CA, USA) was subsequently used solely to improve language quality, simplify and reconstruct sentences, and enhance the clarity and readability of the manuscript, including assisting in the presentation of complex mechanistic concepts in a more accessible manner for readers. Figure 1, Figure 2, Figure 3, Figure 4 and Figure 5 were primarily prepared and assembled by the authors using Microsoft PowerPoint, with partial assistance from BioRender under the authors’ licensed subscription. Figure 6 was initially developed using FigureLabs AI under a licensed subscription and subsequently reviewed, scientifically verified, modified, and finalized by the authors in Microsoft PowerPoint. The authors take full responsibility for the scientific accuracy and final content of all figures.

## 2. Curcumin, Tumor-Associated Macrophages, and the Molecular Basis for Nanodelivery

The pharmacological and immunological arguments for curcumin nanodelivery converge on one principle: biological activity depends on exposure at the relevant cellular site. Native curcumin’s limited aqueous solubility and rapid metabolism have been documented for two decades [1,2,3,4,5,6,7]. In clinical pharmacokinetic studies, the circulating parent compound is typically low relative to the doses administered, while conjugated metabolites predominate [3,4,5]. Modern formulations can increase measured systemic exposure, but ‘bioavailability’ is not synonymous with delivery to a particular macrophage population. For an immunonanomedicine, the relevant chain is formulation stability in blood, tumor access, interstitial transport, interaction with the mononuclear phagocyte system, cell-type-specific uptake, intracellular release, and persistence of pharmacologically active curcumin long enough to alter signaling. Each step can fail independently.

The second foundation is macrophage plasticity. Macrophages in tumors arise from both recruited monocytes and, depending on tissue, embryonically seeded resident populations [18,57,58]. Tumor-derived CCL2 can recruit inflammatory monocytes, while CSF1 and other factors shape survival and differentiation [29,30,31,32]. Human transcriptomic and pan-cancer immune-infiltration studies show that macrophage abundance and TAM programs are clinically relevant yet cancer-specific, with states enriched for antigen presentation, lipid handling, phagocytosis, interferon response, angiogenesis, matrix remodeling, or SPP1-associated programs [24,25,26,27,59]. This heterogeneity is central to delivery design: a ligand selected against IL-4-polarized murine macrophages does not automatically recognize the dominant suppressive macrophage state in a human tumor, and a carrier preferentially engulfed by phagocytes may reach both tumor-promoting and tumor-restraining macrophage subsets.

Within this complexity, classical macrophage-polarization literature still provides a useful mechanistic scaffold, provided that it is not mistaken for a complete description of human TAM states [60]. Several recurring molecular axes remain useful. IL-4/IL-13-JAK-STAT6 signaling supports alternative activation programs; IL-6/IL-10-STAT3 signaling can reinforce immunosuppressive transcription; PI3Kγ acts as a myeloid molecular switch linked to checkpoint resistance; hypoxia and lactate modify macrophage state; and TGF-β can contribute to T-cell exclusion [28,35,36,61,62,63]. Conversely, STAT1-associated programs, IL-12 production, inducible nitric oxide synthase, costimulatory molecules, and chemokines that recruit effector lymphocytes characterize many experimentally defined antitumor macrophage states. These axes are not mutually exclusive and should not be treated as an invariant M1/M2 wiring diagram. A macrophage can simultaneously express inflammatory and tissue-remodeling modules, and pathway direction can change with dose, timing, cell origin, and local cytokine context.

This distinction is especially important for curcumin. In tumor models, curcumin has been reported to reduce M2-associated markers, suppress STAT3/STAT6-related programs, alter cytokine output, and restore antitumor functions [64,65,66,67,68]. Yet curcumin can promote M2-like anti-inflammatory polarization in non-cancer inflammatory disease. Thus, the molecule is not intrinsically a universal ‘M1 polarizer’. It is better understood as a context-sensitive network modulator whose net macrophage phenotype reflects baseline activation state, extracellular signals, concentration, intracellular exposure, and the pathways that dominate in that model. This is precisely why nanodelivery should be evaluated as a pharmacological variable rather than as a neutral packaging step.

### 2.1. Curcumin Pharmacology and the Delivery Barrier

For a molecular review, a major weakness of the conventional curcumin literature is the disconnect between nominal dose, intracellular parent-curcumin exposure, and the pharmacodynamic pathway changes attributed to that exposure. In vitro studies frequently use micromolar concentrations that can be maintained in culture medium, whereas oral pharmacokinetic studies show extensive glucuronidation, sulfation, reduction, and clearance [2,3,4,5,6]. Chemical instability further complicates interpretation because decomposition products can contribute to measured biological effects. Nanocarriers can protect curcumin from degradation, increase apparent solubility, delay release, or alter the fraction transported to tumors [1,6,11]. However, an improved plasma area under the curve does not prove that the active parent compound entered TAMs; conversely, a carrier avidly taken up by phagocytes may achieve meaningful macrophage exposure even without a dramatic increase in total tumor concentration.

This suggests three exposure metrics that should be separated in future studies. The first is systemic exposure: parent curcumin and major metabolites in plasma over time. The second is tumor exposure: total and free curcumin in tumor tissue, preferably measured by validated liquid chromatography–mass spectrometry (LC-MS) methods. The third is cell-resolved exposure: curcumin or a traceable surrogate quantified in sorted TAMs, tumor cells, dendritic cells, and other relevant populations. The third metric is largely missing from the field. Fluorescent carrier localization can show where a nanoparticle travels, but it is not equivalent to measuring released curcumin in the same cell. Likewise, whole-tumor fluorescence can be dominated by extracellular or phagolysosomal signal. This gap is central because signaling claims depend on intracellular pharmacology, not merely on nanoparticle presence.

Applying these distinctions to the principal nanodelivery studies reveals substantial heterogeneity in what was actually measured. The M2pep-chitosan-Cur system had a mean hydrodynamic diameter of 182 ± 15.3 nm, with approximately 84% drug encapsulation efficiency and 5.6% loading capacity; Cur release during the first 2 h was approximately 20%, 30%, and 40% at pH 7.4, 6.5, and 5.0, respectively [40]. Macrophage uptake was demonstrated by fluorescence imaging and flow cytometry, but these measurements did not quantify released parent Cur within TAMs. In the dual pH-sensitive Cur/anti-PD-1 nanodrug, hydrodynamic diameter decreased from approximately 50 nm at pH 7.4 to 43 nm at pH 6.5; approximately 70% of anti-PD-1 was released within 15 h at pH 6.5, whereas Cur release was only 12.7% after 48 h at pH 7.4 and became markedly faster under lysosome-like acidic conditions [43]. In the PD1-nanovesicle-coated Cur-PLGA system, the Cur-PLGA core measured 149.4 ± 1.5 nm and the final PD1@Cur-PLGA construct measured 177 ± 5.7 nm; therapeutic experiments used intravenous administration at a Cur-equivalent dose of 4 mg/kg [45]. In vivo biodistribution was assessed using DiR-labelled nanoparticles, providing carrier-associated fluorescence rather than quantitative measurement of parent Cur in tumor or immune-cell compartments. These examples illustrate why physicochemical characterization, release behavior, carrier localization, and intracellular parent-drug exposure should be treated as distinct pharmaceutical evidence rather than interchangeable indicators of successful delivery.

The pharmacokinetic problem also shapes dose–response interpretation. Curcumin can suppress proliferation, induce cell death, or alter redox status at higher concentrations, while lower exposures may primarily affect transcriptional or cytokine programs. The ovarian cancer study using patient-ascites TAMs is informative in this regard: low-dose curcumin altered macrophage polarization and conditioned-medium effects at concentrations that were not directly cytotoxic to the cancer cells [67]. Such separation is valuable because it allows immune-mediated effects to be distinguished from nonspecific tumor cell killing. Delivery studies should adopt the same logic by testing whether a nanocarrier shifts the concentration–response relationship for macrophage signaling at exposures achievable in vivo.

### 2.2. Molecular Programming of TAMs

The dominant macrophage program in a tumor reflects recruitment, ontogeny, spatial location, and continuous sensing of cytokines, metabolites, extracellular matrix, hypoxia, dying cells, and therapy-induced signals. CCL2-CCR2-dependent recruitment and CSF1-CSF1R survival are well-established components of this ecology [29,30,31,32,33]. Once inside the tumor, macrophages can be pushed toward immunosuppressive functions by IL-10, TGF-β, hypoxia, lactate, and PI3Kγ-dependent signaling [28,34,35,36,61]. These programs can reduce IL-12 production, support angiogenesis, enhance tumor-cell invasion, and interfere with the recruitment or function of cytotoxic lymphocytes.

STAT3 and STAT6 provide useful molecular anchors because both appear repeatedly in curcumin-TAM studies. STAT6 is activated downstream of IL-4/IL-13 and contributes to the transcription of *Arg1*, *Mrc1*/CD206, and other alternative-activation genes in experimental macrophages. Curcumin-mediated inhibition of the MAO-A/STAT6 axis reduced CD206, Arg1, IL-10, and TGF-β while increasing CD86, iNOS, and inflammatory cytokines in oral-cancer-conditioned or IL-4-polarized macrophages [64]. STAT3 integrates IL-6/IL-10 and tumor-derived signals and is linked to immunosuppressive myeloid states. In breast-cancer models, curcumin reduced PI3K-AKT/STAT3 activity and TGF-β production in M2-like macrophages, thereby limiting macrophage-mediated paclitaxel resistance [68]. Related studies identify STAT3/IL-10 loops as macrophage-tumor interaction nodes independent of curcumin [69,70,71], supporting the biological plausibility of this pathway while not converting those studies into direct evidence of curcumin action.

NF-κB requires more careful treatment. Curcumin was originally characterized as a suppressor of NF-κB activation in cancer-associated contexts [9], and several modern nanodrug designs exploit inhibition of NF-κB-associated inflammatory or survival pathways. Yet antitumor macrophage activation can itself require NF-κB-dependent inflammatory transcription. In glioblastoma models, parent-Cur formulations have been associated with increased NF-κB/STAT1-related antitumor microglial activity [51,52], whereas a dual pH-sensitive Cur nanodrug used NF-κB inhibition as part of a strategy to relieve immunosuppression [43]. The defensible conclusion is therefore network- and cell-state-specific: the relevant question is which NF-κB program is operating in which cell, at what time, and with what functional consequence. Recent studies outside the tumor setting further illustrate this context dependence. Sclerostin–ApoER2 signaling suppressed NF-κB activity and promoted anti-inflammatory macrophage phenotypes in cardiovascular models, whereas the antimicrobial peptide BSN-37 enhanced macrophage activation through a circBptf–Rbm14-associated nuclear regulatory module involving cytokine and antigen-presentation-related programs [72,73]. These studies do not constitute direct Cur–TAM evidence, but they reinforce that macrophage immune function is governed by stimulus-, tissue-, and disease-specific regulatory circuits rather than by a universal activation program.

Hypoxia and metabolism further diversify TAM states. Tumor-derived lactate can promote a protumor macrophage program, and macrophage HIF-1α can suppress T-cell function [28,61]. HIF-1α also directly induces PD-L1 under hypoxia [74]. These findings create a mechanistic bridge to curcumin nanoplatforms designed around photodynamic or redox-responsive therapy, where oxygen consumption, ROS generation, and hypoxic signaling are not secondary details but determinants of immune outcome. A curcumin-loaded upconversion platform, for example, was developed to combine photodynamic activity with inhibition of HIF-1α-associated immunosuppression and TAM reprogramming [42]. This is a stronger molecular rationale than the generic claim that phototherapy ‘activates immunity’. Additional tumor-microenvironment work suggests that the inflammatory context and tumor genotype can modify the response to curcumin, reinforcing the need to avoid a one-directional polarization model [66]. Figure 2 summarizes how macrophage origin and tumor-microenvironmental inputs generate heterogeneous, plastic TAM states with distinct functional consequences. This biological context is used throughout the review to interpret Cur-associated macrophage changes without reducing TAM biology to a binary M1/M2 model.

## 3. Engineering Curcumin Nanodelivery Toward TAM-Rich Tumor Microenvironments

Nanodelivery strategies relevant to TAMs fall into four mechanistically distinct categories: (i) passive or tumor-accumulating systems that improve curcumin exposure but do not possess a macrophage-specific targeting element; (ii) stimulus-responsive systems in which pH, redox state, ROS, ultrasound, heat, or other tumor-associated cues trigger release; (iii) actively targeted systems that use a ligand with experimentally demonstrated affinity for a macrophage population; and (iv) biomimetic or immune-integrated systems that use cell membranes, engineered vesicles, antibodies, or checkpoint-binding components. These categories should not be conflated. A macrophage-membrane-coated particle may use macrophage membrane proteins to home to inflamed tumor tissue, but that does not necessarily mean the particle targets TAMs. Conversely, an M2pep-modified carrier is explicitly designed to bind an experimentally defined M2-like macrophage population [40,75,76,77,78,79,80,81].

The physical constraints of solid tumors are equally important. The classical enhanced permeability and retention concept explains part of macromolecular tumor accumulation [82], but quantitative analyses show that only a small fraction of administered nanoparticles reaches many tumors, and entry can occur through multiple vascular transport mechanisms rather than a single leaky-vessel route [83,84]. Size, shape, surface chemistry, protein corona, tissue perfusion, interstitial pressure, and phagocyte capture all influence distribution [85,86,87,88,89,90,91,92]. For TAM-directed delivery, capture by the mononuclear phagocyte system can be either a liability or an opportunity. Off-target hepatic and splenic macrophage uptake may reduce tumor delivery, whereas controlled recognition by tumor phagocytes may increase local drug exposure. The engineering objective is therefore selective tissue and cell engagement, not maximal phagocytosis everywhere.

This framework is particularly useful for evaluating curcumin studies. Several reports show antitumor efficacy and immune remodeling without directly measuring curcumin concentration in TAMs [41,42,43,44,45,46,47,48,49]. These should be described as delivery-assisted or immune-remodeling systems rather than as macrophage-targeted curcumin unless the targeting claim is experimentally demonstrated. In contrast, the M2pep-chitosan-curcumin system provides a clearer macrophage-targeting logic and includes anti-PD-L1 combination testing [40]. The distinction is not semantic; it determines what can be claimed about the mechanism and what must remain an inference.

### 3.1. Passive, Stimuli-Responsive, and Tumor-Microenvironment-Activated Curcumin Delivery

Stimuli-responsive systems attempt to solve two competing problems: curcumin should remain associated with the carrier during circulation but become available after the carrier reaches a relevant tumor compartment. Acidic pH is the most frequently exploited cue, reflecting extracellular acidification and the endosomal/lysosomal environment. Redox-sensitive and ROS-responsive linkers provide complementary release mechanisms, whereas light, heat, and ultrasound permit externally controlled activation. The dual pH-sensitive curcumin/checkpoint nanodrug and the inflammation-regulated HCC nanodrug illustrate how release behavior can be integrated with immune signaling rather than treated as a purely pharmaceutical feature [43,44]. In both cases, the immune effect depends on where the drug is released and which cell populations encounter the released cargo.

Photodynamic and photothermal designs introduce additional molecular complexity. Curcumin can act as a photosensitizing or redox-active component, while phototherapy can generate ROS, damage tumor cells, expose damage-associated molecular patterns, alter perfusion, and intensify hypoxia. The core-satellite upconversion system explicitly addressed HIF-1α and TAM polarization after photodynamic therapy, providing a mechanistic example of how delivery and microenvironmental feedback can be coupled [42]. More recent photothermal-responsive copper-based nanocomposites add checkpoint therapy to this logic [46]. These systems are attractive because they can generate a local immune perturbation, but they are also difficult to interpret: tumor-cell death, ROS, heat, curcumin signaling, carrier effects, and checkpoint blockade can all contribute. Factorial controls are therefore essential.

Ultrasound-responsive nanobubbles and ROS-responsive conjugates represent another step toward conditional release. The curcumin/miR-16-5p nanobubble platform couples ultrasound-triggered delivery with ICD and PD-L1 suppression in HCC [48], while an anti-PD-L1-curcumin conjugate uses a ROS-responsive linker to combine tumor targeting and local drug release [47]. These are not TAM-specific systems, yet they are directly relevant to the review because they show how curcumin delivery can be engineered to perturb the same immune-checkpoint network that macrophage reprogramming is expected to influence. The remaining challenge is to resolve the cellular path: does curcumin act principally in tumor cells, TAMs, dendritic cells, or several compartments simultaneously?

### 3.2. Active and Biomimetic Strategies for Macrophage-Directed Curcumin Delivery

M2pep provides the clearest experimental precedent for active TAM-directed delivery. The peptide was identified for preferential binding to murine M2-like macrophages and was subsequently used to deliver proapoptotic cargo, PLX3397, and CSF1R-targeted siRNA [75,76,77]. These studies establish an engineering principle: macrophage-selective ligands can increase local engagement and amplify the immunological effect of a cargo. In the curcumin field, M2pep-chitosan-curcumin nanoparticles extend this principle to a natural-product payload and provide direct evidence of macrophage uptake and TAM reprogramming in TNBC [40]. Mechanistically, COX-2 overexpression in M2 macrophages opposed the NP-associated macrophage-state shift and increased proliferation, migration, and invasion of co-cultured MDA-MB-231 and BT-549 cells, providing perturbational evidence that COX-2 contributes to the macrophage-mediated effect [40]. In vivo, the nanoparticle-plus-anti-PD-L1 group showed greater tumor suppression than the nanoparticle-only group; however, the absence of an anti-PD-L1-monotherapy arm prevents independent attribution of this incremental benefit to checkpoint sensitization or formal synergy. Moreover, although TAM reprogramming accompanied treatment, no macrophage-depletion, blocking, or rescue experiment demonstrated that the reprogrammed macrophage state was necessary for the combination benefit. The study by Dong et al. [40] should therefore be regarded as strong evidence for TAM-associated curcumin delivery and reprogramming, but only suggestive evidence for TAM-mediated enhancement of checkpoint therapy. M2pep was developed against defined murine macrophage states; its binding spectrum across human TAM states remains an important translational question.

Mannose/CD206-directed strategies are also attractive because CD206 is commonly enriched in macrophages described as M2-like. However, CD206 is neither unique to tumor-promoting macrophages nor uniformly expressed across human TAM populations. Mannosylated R848 nanoparticles can target macrophage and dendritic-cell compartments and convert a cold tumor toward a more inflamed immune state [80]. Biomimetic mannosylated albumin nanoparticles and related systems show how tumor-cell and macrophage targeting can be combined [81]. These studies are most useful here as engineering precedents: they identify ligand density, receptor-mediated uptake, cell selectivity, and immunological readouts that should be incorporated when adapting such platforms to curcumin.

Macrophage-membrane coating represents a different strategy. In the Cur-PDA@CM platform, the macrophage membrane was used to enhance tumor homing, while the delivered curcumin/polydopamine system repolarized TAMs and supported photothermal therapy [41]. The important semantic distinction is that the coating is macrophage-derived, but the target is primarily the tumor tissue; the TAM effect is downstream. Engineered cell-derived nanovesicles can similarly confer tumor-homing or checkpoint-binding functions. PD-1-overexpressing nanovesicles coating curcumin-loaded PLGA nanoparticles combine PD-1/PD-L1 blockade with curcumin-mediated ICD, increasing dendritic-cell maturation and CD8^+^ T-cell infiltration [45]. These carriers combine targeting and immunological functions but introduce additional challenges in manufacturing, compositional characterization, and batch-to-batch control.

### 3.3. Engineering Lessons from TAM-Targeted Nanomedicine Beyond Curcumin

The broader TAM nanomedicine literature can guide curcumin platform design without being misrepresented as curcumin evidence. Three lessons are particularly transferable. First, cell-type specificity must be demonstrated quantitatively. M2pep, dual M2pep/SR-B1 targeting, and mannosylated carriers have been evaluated with comparative uptake in macrophage subsets and non-target tissues [75,76,77,80,81]. Curcumin systems should adopt the same standard rather than inferring TAM targeting from whole-tumor fluorescence. Accordingly, this review considers TAM targeting to be directly demonstrated when cell-resolved uptake is shown in phenotypically defined TAMs and preferential uptake is quantitatively established relative to relevant tumor cells or other stromal/immune populations. For ligand- or receptor-directed systems, receptor dependence should additionally be supported by blocking, competition, knockdown, or targeted-versus-untargeted controls; simple whole-tumor accumulation or qualitative colocalization alone is considered supportive rather than definitive evidence of TAM targeting [75,76,77,80,81]. TAM uptake and TAM reprogramming are evaluated separately: cell-resolved nanoparticle uptake demonstrates exposure, whereas reprogramming requires independent evidence of altered macrophage state or function, such as changes in transcriptional programs, cytokine output, phagocytosis, antigen presentation, metabolic activity, or functional effects on other immune cells. Uptake alone is therefore not considered evidence of macrophage reprogramming.

Second, the cargo mechanism should be orthogonal to the targeting mechanism. R848-loaded cyclodextrin nanoparticles reprogrammed TAMs through TLR7/8 signaling and improved anti-PD-1 response [78]; CSF1R-directed siRNA changed macrophage survival/state through a defined molecular target [77]. This makes it easier to attribute the immune effect to a known pathway.

Third, TAM-directed nanomedicine increasingly measures downstream adaptive immunity. The most informative studies do not stop at CD86/CD206 ratios; they quantify cytokines, CD8^+^ T-cell infiltration, T-cell exhaustion markers, dendritic-cell behavior, tumor rechallenge, or checkpoint combination efficacy [77,78,80,81]. Curcumin nanodelivery should be held to the same standard. A formulation that shifts macrophage markers but does not alter macrophage function or the immune network has demonstrated phenotype modulation, not checkpoint sensitization. Conversely, a platform that enhances anti-PD-1 efficacy without measuring macrophage engagement has demonstrated combination benefit but not a TAM-mediated mechanism. The review therefore evaluates these findings as distinct evidence domains rather than allowing evidence in one domain to substitute for evidence that was not measured in another. Figure 3 compares major Cur delivery architectures and distinguishes validated TAM-directed uptake from passive tumor accumulation, secondary TAM exposure, biomimetic tumor homing, and checkpoint-integrated delivery. The evidence summarized in Table 1 is used here to separate formulation performance from macrophage-specific delivery and uptake, intracellular exposure, pharmacodynamic pathway modulation, functional reprogramming, and downstream immune effects.

The evidence summarized in Table 1 is used here to separate formulation performance from demonstrated macrophage-specific uptake/exposure, molecular reprogramming, and downstream immune effects. Table 2 complements this direct curcumin evidence by summarizing TAM-targeted nanomedicine precedents with non-curcumin cargos that provide transferable engineering principles for ligand selection, cell-specific uptake, pathway engagement, and immune-function validation.

## 4. Molecular Pathways Underlying Curcumin-Mediated TAM Reprogramming

The molecular evidence for curcumin-mediated macrophage reprogramming is strongest when pathway measurements are connected to functional macrophage outputs rather than inferred from docking or marker changes alone. Direct studies identify STAT6, STAT3, PI3K-AKT, HIF-1α, NF-κB-associated networks, cytokine circuits, and tumor-cell/macrophage feedback as recurrent nodes [42,43,44,64,65,66,67,68]. The pattern is not a single linear pathway. Curcumin acts as a network-active molecule, and nanodelivery changes the amplitude, duration, and cellular compartment of that network perturbation. Accordingly, the mechanistic focus should be the pathway that is rate-limiting in the targeted cell state at the delivered exposure rather than the assumption of a single universally inhibited pathway.

Throughout this review, three mechanistic evidence categories are distinguished. Pharmacodynamic pathway modulation refers to treatment-associated changes in signaling or molecular readouts, such as phosphorylation of STAT3, STAT6, or AKT, NF-κB activity, or HIF-1α abundance. Causal pathway attribution requires additional perturbation evidence, such as genetic knockdown, pathway-selective inhibition, or rescue demonstrating that the altered pathway contributes to the observed phenotype. Direct molecular target engagement is used more narrowly for evidence that curcumin physically interacts with or occupies a defined molecular target, established using an appropriate binding, occupancy, biophysical, chemoproteomic, or related target-engagement assay. Accordingly, changes in pathway activity alone are not interpreted as evidence of direct curcumin–protein binding [94,95].

Evidence outside curcumin reinforces this systems view. PI3Kγ can function as a myeloid switch controlling immune suppression and checkpoint resistance [35,36]; hypoxia/HIF-1α can suppress T-cell function and induce PD-L1 [61,74]; and STAT3/IL-10 communication can stabilize macrophage-centered immunosuppressive circuits [69,70,71]. These independent studies are mechanistically supportive because they validate the biological importance of the nodes that curcumin studies perturb. They do not prove that every curcumin formulation reaches those nodes in vivo. That distinction is retained throughout the pathway synthesis.

### 4.1. STAT3/STAT6 Networks

STAT6 is most directly implicated in experimental curcumin-driven macrophage repolarization through the MAO-A/STAT6 study. In tumor-conditioned or IL-4-stimulated macrophages, curcumin reduced STAT6-associated protumor markers while increasing an antitumor-like cytokine and surface-marker profile. The strength of this evidence is that pathway modulation and phenotype were measured in the same system. Its limitation is that the macrophage state was simplified relative to a human tumor, and the observed STAT6 pathway changes do not establish direct curcumin–STAT6 binding or target occupancy. The study therefore supports STAT6-associated pharmacodynamic modulation and functional macrophage reprogramming [64], but should not be interpreted as evidence that curcumin directly engages STAT6 as a molecular target.

STAT3 evidence is broader. The historical hydrazinocurcumin nanoparticle study reported STAT3 suppression together with TAM re-education and antitumor activity [54]; however, a subsequent Expression of Concern identified unresolved concerns regarding control and image data, substantially limiting the evidentiary weight that should be assigned to this study [55]. More recent evidence comes from distinct experimental contexts. Curcumin suppressed PI3K-AKT/STAT3 signaling and TGF-β output in M2-like macrophages that otherwise promoted paclitaxel resistance in breast-cancer cells [68]. In LUAD, combined computational and experimental analyses linked curcumin treatment to reduced CCNA2 expression, modulation of AKT/STAT signaling, and reduced macrophage M2 polarization [96]. In anaplastic thyroid carcinoma, curcumin reduced AKT/mTORC1/STAT3/PD-L1 signaling while enhancing CD8^+^ T-cell function [97]. Collectively, these studies support STAT3-associated signaling as a recurrent curcumin-responsive network across macrophage and tumor-cell compartments, while the upstream mechanisms and cellular context differ substantially among models.

The translational implication is that a STAT3-centric nanoparticle should measure pharmacodynamic pathway modulation in the intended cell population. Whole-tumor pSTAT3 reduction is insufficient because tumor cells, fibroblasts, endothelial cells, and immune cells can all contribute. An informative study would quantify parent curcumin in sorted TAMs, measure pSTAT3/pSTAT6 and downstream transcription in the same cells, and demonstrate functional macrophage reprogramming. Genetic or pharmacological perturbation and rescue can then strengthen causal attribution of the pathway to the phenotype. Direct curcumin–protein target engagement, however, would require a separate assay capable of demonstrating physical interaction or target occupancy.

### 4.2. NF-κB, PI3K/AKT and MAPK Networks

NF-κB illustrates why curcumin-TAM biology cannot be reduced to a universal inhibition model. Curcumin can suppress NF-κB activation in tumor and inflammatory systems [9], and NF-κB inhibition is incorporated into some curcumin nanodrug designs [43,44]. Yet macrophage antitumor activity can require inflammatory NF-κB-dependent transcription, while glioblastoma studies of curcumin formulations reported restoration of an activated microglial/macrophage phenotype associated with STAT1/NF-κB-linked functions [51,52]. The available evidence requires compartment-specific interpretation of NF-κB signaling: tumor-cell NF-κB that supports survival or PD-L1-linked inflammation; suppressive TAM NF-κB programs associated with chronic tumor-promoting inflammation; and acute innate NF-κB programs required for microbial-like or TLR-driven antitumor activation. Curcumin may affect each differently. Recent breast-cancer/macrophage experiments also implicate the NLRP3/caspase-1/IL-1β inflammasome axis as a curcumin-sensitive inflammatory node, although this pathway should be treated as complementary to, rather than synonymous with, NF-κB signaling [98].

PI3K-AKT has a more consistent relationship with immunosuppressive myeloid signaling. Genetic and pharmacological studies identify PI3Kγ as a switch that promotes suppressive transcription and resistance to checkpoint blockade [35,36]. Curcumin inhibited PI3K-AKT/STAT3 signaling in M2 macrophages in the paclitaxel-resistance model [68]; LUAD experiments associated reduced CCNA2 expression with AKT/STAT modulation and reduced macrophage M2 polarization [96]; and anaplastic thyroid carcinoma experiments linked curcumin treatment to suppression of the AKT/mTORC1/STAT3/PD-L1 axis with enhanced CD8^+^ T-cell function [97]. These data justify a pathway module in which nanodelivery increases intracellular curcumin and thereby changes PI3K-AKT-dependent signal integration. The claim should remain conditional, however, because not every curcumin nanoparticle study measured AKT in TAMs.

MAPK signaling is often invoked in curcumin reviews but is less directly resolved in the curcumin-TAM literature. ERK, JNK, and p38 can affect cytokine production, differentiation, stress signaling, and cell death, yet broad MAPK changes after curcumin exposure do not establish a macrophage-specific causal mechanism. For this review, MAPK is therefore treated as a mechanistically supportive network rather than a central proven axis. Future nanodelivery studies should use time-resolved phosphoproteomics or targeted kinase assays to determine whether MAPK changes precede macrophage state transitions or merely accompany cell stress. This is an example of evidence discipline: plausible biology should not be promoted to direct proof because it fits a familiar pathway diagram.

### 4.3. Hypoxia, Redox and Metabolic Programming

Hypoxia, redox state, and metabolism are tightly coupled in TAM biology. Lactate can polarize TAMs toward tumor-supporting functions [28], macrophage HIF-1α can suppress T-cell responses [61], and HIF-1α can directly induce PD-L1 [74]. These pathways become particularly relevant when curcumin is delivered in photodynamic, photothermal, or ROS-responsive systems. The Cur-CSNP platform was designed around this feedback: photodynamic therapy can exacerbate hypoxia, whereas curcumin-mediated HIF-1α suppression was used to counter an immunosuppressive rebound while promoting TAM repolarization and lymphocyte infiltration [42]. This is a strong example of delivery architecture being chosen in response to a molecular liability created by the therapy itself. Independent HCC experiments further show that curcumin can decrease STAT3/VEGF/HIF-1α signaling in tumor cells [99]; this supports pathway plausibility but does not establish TAM-specific HIF-1α modulation, causal pathway dependence, or direct curcumin–HIF-1α binding.

Redox biology is similarly bidirectional. Curcumin is often described as an antioxidant, yet in cancer cells and phototherapy systems, it can participate in pro-oxidant stress, ROS generation, mitochondrial dysfunction, and ICD. Cur-PDA@CM nanoparticles increased intracellular ROS while remodeling TAM polarization [41], whereas curcumin–doxorubicin nanoparticles increased ROS and ICD-associated markers, including CRT exposure, HMGB1 redistribution, and ATP release, without directly establishing the proposed ER-stress signaling mechanism [93]. The biological outcome therefore depends on dose and compartment: transient redox modulation in macrophages may alter signaling, whereas stronger oxidative stress in tumor cells may drive death and release of danger signals that secondarily activate antigen-presenting cells.

Metabolic state should be incorporated more directly into future TAM-curcumin studies. Human single-cell datasets identify macrophage states linked to lipid handling, hypoxia, phagocytosis, and SPP1-associated matrix remodeling [24,25,26,27]. Tetrahydrocurcumin, which is chemically distinct from parent Cur, has been reported to modulate the SPP1/CD44 axis and reduce M2 polarization in colorectal cancer [56]. This study is used as supportive curcuminoid evidence and not as direct evidence for parent-Cur nanodelivery. The next generation of curcumin immunonanomedicine should move beyond static CD206/Arg1 measurements toward metabolic flux, lipidomics, mitochondrial function, hypoxia sensing, and spatial transcriptomics. Figure 4 integrates the molecular pathways with the level of evidence supporting each curcumin-TAM connection, avoiding the common assumption that every plausible pathway has been experimentally validated.

## 5. From TAM Reprogramming to Immune-Checkpoint Sensitization

Checkpoint sensitization is a stronger claim than immune modulation. To demonstrate sensitization, a study should show that curcumin or its delivery system changes a resistance mechanism and improves the therapeutic effect of PD-1/PD-L1 blockade beyond either component alone. TAM biology provides several routes by which this could occur. Macrophage IL-10 can suppress dendritic-cell IL-12 and weaken CD8^+^ T-cell responses [34]; macrophages can restrict T-cell access to tumor nests [37]; PD-1-expressing TAMs can show impaired phagocytosis [38]; and TAMs can mediate antibody redistribution or resistance during anti-PD-1 therapy [39]. At the same time, TGF-β, hypoxia, and PI3Kγ-dependent myeloid programs contribute to T-cell exclusion or checkpoint resistance [35,36,62,63,74]. A curcumin platform that reprograms these pathways could therefore sensitize checkpoint therapy even if curcumin does not bind PD-1 or PD-L1 directly.

The direct curcumin evidence now spans several architectures. In HCC, curcumin combined with anti-PD-1 produced greater antitumor effects than either agent alone and was linked to reduced PD-L1/TGF-β-associated immune suppression [100]. A dual pH-sensitive nanodrug combined curcumin-mediated NF-κB inhibition with PD-1 blockade [43], while an inflammation-regulated curcumin/anti-PD-L1 nanodrug reprogrammed the HCC microenvironment [44]. In TNBC, M2pep-chitosan-curcumin nanoparticles promoted macrophage uptake and TAM reprogramming, and the nanoparticle-plus-anti-PD-L1 group exhibited greater tumor suppression than nanoparticles alone [40]. Nevertheless, because anti-PD-L1 monotherapy was not included in the in vivo comparison and macrophage dependence of the combination effect was not experimentally tested, the study does not establish checkpoint sensitization, formal synergy, or the necessity of TAM reprogramming for the additional therapeutic benefit. It therefore supports several components of the proposed mechanistic chain without demonstrating the complete delivery–TAM–checkpoint causal sequence.

Checkpoint-related nanoplatforms should also be distinguished according to experimental architecture rather than treated as interchangeable evidence. The dual pH-sensitive Cur/anti-PD-1 nanodrug was evaluated in immunocompetent B16F10/C57BL/6 mice using free Cur, free anti-PD-1, Cur-only nanocarrier, antibody-bearing carrier, and dual-drug nanocarrier comparators, providing comparatively strong component-level control of the checkpoint contribution [43]. Other systems represent different evidentiary categories: the inflammation-regulated platform co-delivers Cur and anti-PD-L1 within a single nanodrug [44]; PD1@Cur-PLGA employs PD1-overexpressing nanovesicles as an engineered checkpoint-binding carrier [45]; the photothermal Cur nanocomposite is evaluated together with separately administered anti-PD-1 [46]; and the anti-PD-L1-PBA-Cur construct is an antibody–drug conjugate rather than a conventional checkpoint-combination experiment [47]. By contrast, the Cur/miR-16-5p nanobubble system does not administer a checkpoint inhibitor, because miR-16-5p is used to suppress PD-L1 while Cur contributes to ultrasound-triggered immunogenic cell death [48]. These designs therefore provide different forms of evidence and should not be interpreted equivalently. Combination benefit is distinguished from formally demonstrated synergy, while reversal of checkpoint resistance is reserved for studies in which an established resistant state is prospectively demonstrated and subsequently reversed.

Other systems approach the checkpoint axis through tumor-cell PD-L1 regulation or ICD. PD1-decorated Cur-PLGA nanoparticles combine ligand-level blockade with curcumin-induced ICD and increase mature dendritic cells and CD8^+^ T-cell infiltration [45]. Photothermal-responsive curcumin nanocomposites combine local therapy with anti-PD-1 [46]. Anti-PD-L1-conjugated curcumin uses a ROS-responsive linker and reduces tumor-surface PD-L1 [47]. Curcumin/miR-16-5p nanobubbles couple ICD induction to PD-L1 suppression [48]. These platforms demonstrate checkpoint-integrated curcumin delivery, but macrophage mediation varies. They should therefore be positioned as near-direct evidence for the broader review axis, not as proof that TAM reprogramming is the universal route of sensitization.

Non-nano studies add mechanistic support. Curcumin reduced PD-L1 and enhanced CD8^+^ T-cell function through AKT/mTORC1/STAT3 signaling in anaplastic thyroid carcinoma [97]; it reduced IFN-γ-induced PD-L1 through STAT1-related signaling in A549 cells [101]; and it enhanced T-cell-mediated killing in colorectal cancer through a miR-206/JAK/STAT3/PD-L1-associated mechanism [102]. Curcumin also modifies suppressive stromal circuits: inhibition of CAF-derived TGF-β reduced PD-L1 and improved anti-PD-1 therapy in colorectal cancer [103]. These findings broaden the concept from a macrophage-only model to a network model in which curcumin may act on tumor cells, macrophages, fibroblasts, dendritic cells, and lymphocytes simultaneously.

PD-1/PD-L1 signaling itself should be represented at molecular resolution. PD-1 engagement recruits phosphatases and preferentially suppresses CD28 costimulatory signaling; effective rescue of exhausted T cells by PD-1-targeted therapy is CD28-dependent [104,105,106]. Thus, improved CD8^+^ infiltration alone does not prove restored function. Stronger studies should quantify IFN-γ, granzyme B, proliferation, degranulation, exhaustion markers, and, where possible, antigen-specific killing. The Tumor Immune Dysfunction and Exclusion (TIDE) framework and broader resistance literature emphasize that dysfunction and physical exclusion are separable barriers [107,108,109]. TAM reprogramming may address both: it can alter suppressive cytokines and simultaneously change chemokine/spatial conditions that determine whether effector cells reach tumor cells.

ICD provides another bridge. Curcumin-containing systems can enhance calreticulin exposure, HMGB1 release, ATP release, ER stress, dendritic-cell maturation, and downstream cytotoxic immunity [45,48,93]. GSDME-mediated pyroptosis has recently been proposed as a way to potentiate PD-1/PD-L1 blockade in colorectal cancer [110]. These studies suggest that curcumin delivery can increase the antigenic and inflammatory context in which checkpoint blockade operates. Yet ICD must be demonstrated by accepted molecular readouts; increased apoptosis or ROS alone should not be relabeled as ICD. The same evidence discipline applied to TAM targeting must be applied to immunogenicity claims.

### 5.1. TAM-Tumor-Lymphocyte Molecular Crosstalk

TAM reprogramming can influence lymphocytes through several molecular routes. Reduced IL-10 can release dendritic cells from suppression and permit IL-12-dependent CD8^+^ responses [34]. Reduced TGF-β can lessen T-cell exclusion and suppressive fibro-myeloid signaling [62,63]. Increased IL-12, TNF, CXCL9/CXCL10, or costimulatory capacity can support effector-cell recruitment and activation, whereas Arg1 and suppressive metabolites can limit nutrient availability. Enhanced antigen presentation and costimulatory signaling can support local T-cell restimulation, while restored macrophage phagocytosis, reduced inhibitory-ligand signaling, and relief of metabolite-mediated suppression can further improve the access, persistence, and effector function of CD8^+^ T cells [34,37,38]. In melanoma, dual-targeted CSF1R-siRNA nanoparticles reduced IL-10/TGF-β, increased IL-12/IFN-γ, and restored CD8^+^ T-cell infiltration and function [77]. R848-loaded TAM-directed nanoparticles similarly converted the immune environment and improved anti-PD-1 response [78]. These non-curcumin studies establish the functional benchmark that curcumin systems should seek. Curcumin also affects suppressive myeloid compartments beyond TAMs: experimental work showed induction of myeloid-derived suppressor-cell differentiation with reduced tumor-promoting interactions [111]. Regulatory T cells represent another potentially curcumin-responsive suppressive compartment, although the supporting literature is broader and less directly tied to TAM-targeted nanodelivery [112].

Curcumin evidence increasingly approaches this benchmark. Studies of parent curcumin using dendrosomal and phytosomal formulations altered macrophage/microglial states and, in the phytosomal GL261 model, supported NK-cell-dependent antitumor effects [50,51,52]. TriCurin also produced TAM/microglial reprogramming, but its three-agent composition prevents attribution of these effects to Cur alone [53]. M2pep-curcumin nanoparticles showed TAM reprogramming together with greater antitumor activity for nanoparticle-plus-anti-PD-L1 treatment than for nanoparticles alone, although checkpoint sensitization and TAM dependence were not independently resolved [40]. BSA-curcumin nanoparticles altered cytokine profiles in an MCF-7/PBMC co-culture system [49], but macrophages were not evaluated; this study therefore provides supportive evidence of in vitro immunomodulation rather than evidence of TAM-specific uptake, macrophage pathway modulation, or functional reprogramming. Patient-derived ovarian TAMs provide a valuable human bridge because low-dose curcumin altered macrophage cytokines and reduced tumor-cell migration/invasion through conditioned-medium effects [67]. Such studies demonstrate functional crosstalk even without checkpoint therapy and should be prioritized in future validation.

### 5.2. PD-1/PD-L1 Regulation and Curcumin-Enabled Checkpoint Sensitization

PD-L1 expression is controlled by constitutive oncogenic pathways and adaptive inflammatory signaling. IFN receptor-JAK/STAT signaling can drive PD-L1/PD-L2 expression [113], while HIF-1α directly induces PD-L1 under hypoxia [74]. Curcumin intersects these routes at several points: STAT3 inhibition can reduce PD-L1 in some tumor cells [97], STAT1 modulation can reduce IFN-γ-induced PD-L1 [101], and HIF-1α-oriented nanotherapy can simultaneously alter hypoxia and macrophage state [42]. The direction of effect is therefore pathway- and context-dependent; a global claim that curcumin ‘blocks PD-L1’ is too broad. Importantly, the direct Cur–PD-L1 evidence considered here is resolved primarily in tumor-cell systems, including A549 lung cancer cells and anaplastic thyroid carcinoma models [97,101]. By contrast, the TAM-focused Cur studies demonstrate macrophage reprogramming or checkpoint-associated effects without directly establishing Cur-mediated regulation of PD-L1 specifically within TAMs. Whole-tumor PD-L1 changes should therefore not be assigned to a macrophage or tumor-cell compartment without cell-resolved analysis; flow cytometry, cell sorting, multiplex imaging, or spatially resolved measurements are needed to distinguish these sources.

For checkpoint combinations, the most persuasive design is mechanistically layered: one arm demonstrates curcumin-dependent molecular change; a second demonstrates immune-cell functional change; and a third demonstrates enhanced checkpoint efficacy. Guo et al. provide non-nano evidence of curcumin/anti-PD-1 synergy in HCC [100]. Dong et al. add TAM-associated curcumin delivery and reprogramming in TNBC together with a checkpoint-combination benefit, although the absence of an anti-PD-L1-only comparator precludes an independent sensitization or synergy claim [40]. Xiao et al., Wang et al., Liao et al., Ji et al., Ding et al., and Xu et al. provide complementary nano-enabled checkpoint strategies [43,44,45,46,47,48]. Together, these studies justify the term ‘checkpoint sensitization’ as an emerging field, but not yet as a clinically established property of nanocurcumin. At a broader combination-therapy level, recent critical analysis has proposed that curcumin-mediated sensitization should be interpreted through mechanistic complementarity, suppression of adaptive resistance, and pharmacokinetic synchronization rather than co-administration alone [114].

A final mechanistic caution concerns treatment order and dose. If a formulation kills tumor cells or macrophages at high concentration, a lower apparent PD-L1 signal may simply reflect cell loss. Likewise, severe oxidative or photothermal injury may recruit immune cells independently of curcumin’s signaling effects. Future combination studies should therefore use viability-normalized PD-L1 measurements, time-resolved pathway assays, and dose matrices that separate immune reprogramming from cytotoxicity. Synergy should be quantified rather than inferred from visually smaller tumors. Figure 5 summarizes the distinct mechanisms by which curcumin delivery may sensitize PD-1/PD-L1 blockade, emphasizing that TAM reprogramming is one route within a broader multicellular network.

## 6. Critical Appraisal of the Experimental Evidence

The literature is now large enough to support a focused review but not sufficiently mature to support a definitive interpretation. Direct evidence that combines all three components—engineered curcumin delivery, TAM-specific uptake/exposure and functional reprogramming, and checkpoint sensitization—remains limited. The strongest studies each cover different segments of the causal chain. Some establish macrophage targeting but provide limited cell-resolved pharmacokinetics and others establish immune-checkpoint synergy but do not prove a macrophage-mediated mechanism; still, others establish molecular reprogramming with free curcumin but not a nanocarrier. The central limitation is the absence of experiments connecting delivery, macrophage function, and checkpoint response within the same model.

A multidimensional evidence framework is therefore more appropriate than a cumulative hierarchy. We evaluate each study independently across eight domains: (D1) pharmaceutical/formulation performance; (D2) tumor delivery/exposure; (D3) TAM-specific uptake and intracellular parent curcumin exposure; (D4) cell-specific pathway modulation; (D5) functional TAM reprogramming; (D6) downstream immune restoration involving dendritic, NK, or T-cell function; (D7) checkpoint-therapy response to PD-1/PD-L1-directed therapy; and (D8) translational or human relevance. For each domain, evidence is classified as directly demonstrated, indirect/supportive, or not measured. These domains are deliberately non-cumulative: demonstration of checkpoint-combination efficacy, for example, does not establish TAM uptake, intracellular curcumin exposure, pathway causality, or macrophage dependence unless those components were independently measured. This structure therefore identifies both the strengths and the missing mechanistic links of each study without assigning an apparently higher evidence grade on the basis of a downstream therapeutic outcome alone.

The commonest experimental weakness is overreliance on simplified macrophage models. RAW264.7 or THP-1-derived macrophages are useful for controlled mechanistic experiments but do not capture the ontogeny, spatial programming, and state diversity revealed by human tumor atlases [24,25,26,27,57,58]. Even primary bone-marrow-derived murine macrophages lack many human tumor-specific cues. Patient-ascites TAM experiments [67] are therefore disproportionately valuable despite their own limitations. Future work should incorporate tumor-derived primary macrophages, humanized models, organoid-myeloid co-cultures, and spatially resolved tissue analysis.

Marker-based polarization is another limitation. A CD86 increase and CD206 decrease can be informative in a defined model, but they are not sufficient to prove an antitumor macrophage function. Functional endpoints should include tumor-cell killing or support, phagocytosis, antigen presentation, cytokine/chemokine secretion, T-cell recruitment, suppression assays, and metabolic behavior. Single-cell RNA sequencing can identify state transitions, but transcriptomic shifts also require functional confirmation. The field should move from ‘M2-to-M1’ as an endpoint toward a multidimensional description of macrophage state and consequence.

Nanodelivery studies face additional attribution problems. A complete control set should include free curcumin, empty carrier, untargeted carrier, targeted carrier without curcumin, and, where possible, a targeting-ligand competition or receptor-blocking control. For multimodal systems, phototherapy, checkpoint antibody, curcumin, and carrier components require factorial comparison. Cell-type-specific uptake should be quantified rather than inferred from tumor accumulation. Finally, molecular causality should be strengthened with genetic or pharmacological rescue. Without these controls, a study may show therapeutic benefit but remain unable to identify which design element caused the immune effect.

Applying these criteria to individual studies reveals substantial differences in mechanistic resolution. In the M2pep-chitosan-Cur study, THP-1-derived macrophages were used for controlled in vitro reprogramming experiments and tumor-associated macrophages were subsequently analyzed; however, the animal-method description is internally inconsistent, identifying C57BL/6J mice in one part of the methods while subsequently describing 4T1 implantation into female nude mice. Immune competence therefore cannot be assigned confidently from the primary report [40]. The dual pH-sensitive Cur/anti-PD-1 study used RAW264.7 macrophages together with an immunocompetent B16F10/C57BL/6 model and incorporated free-drug and single-component nanocarrier controls, but did not demonstrate that macrophages were necessary for therapeutic efficacy through depletion, blockade, or rescue [43]. The PD1@Cur-PLGA study used syngeneic 4T1/BALB/c and RM-1/C57BL/6 models with component controls including free Cur, PD1 nanovesicles, and Cur-PLGA; its immune mechanism was resolved principally through immunogenic cell death, dendritic-cell maturation, and CD8^+^ T-cell responses rather than TAM-dependent causality [45]. In the phytosomal-Cur glioblastoma study, endogenous tumor-associated microglia/macrophages were evaluated in the immunocompetent GL261/C57BL/6 model [52]. NK-cell depletion together with IL-12 blockade provided evidence for NK-cell participation in macrophage-state stabilization and tumor control, but did not establish that macrophages themselves were necessary for the therapeutic effect [52]. The complete study-level comparison of macrophage source, model immune competence, comparator groups, functional assays, checkpoint-monotherapy controls, and immune-cell dependence is provided in Supplementary Table S1.

### 6.1. Multidimensional Evidence-Domain Framework

The framework is intended as a critical-appraisal tool rather than a regulatory grading system or a linear maturity score. Evidence in each domain is assessed independently as directly demonstrated, indirect/supportive, or not measured. Thus, formulation quality does not establish TAM delivery; TAM uptake does not establish functional reprogramming; TAM reprogramming does not establish adaptive immune restoration; and improved outcome with a checkpoint-containing combination does not by itself establish checkpoint sensitization or a TAM-mediated mechanism. Conversely, a study may provide strong checkpoint-combination evidence while leaving upstream TAM-related domains unresolved. The purpose of the framework is therefore to expose these missing links explicitly rather than compress heterogeneous evidence into a single numerical level.

The domain framework also indicates how future studies can strengthen mechanistic continuity without becoming excessively large. A carefully designed experiment can generate evidence across several domains in the same model by quantifying carrier localization, intracellular parent curcumin, pharmacodynamic pathway changes, macrophage function, downstream immune responses, and checkpoint-treatment effects. Importantly, evidence generated in one domain should be reported as such rather than used to fill an unmeasured domain by inference. Such within-study integration is more informative than adding another formulation characterized only by size, zeta potential, release, and tumor-volume data.

### 6.2. Experimental Limitations and Overinterpretation

The curcumin field can also overstate clinical relevance by extrapolating from nominal in vitro concentrations. Concentration–response studies should report free versus carrier-bound drug, protein binding, release kinetics in biologically relevant media, and the actual exposure achieved in tumor tissue and macrophages. Pharmacokinetic comparisons should use parent curcumin and major metabolites. Safety assessment should include liver and spleen macrophage effects, systemic cytokine perturbation, and consequences of repeated dosing. Because macrophages contribute to tissue repair, homeostasis, innate immune surveillance, and host defense, unintended reprogramming of non-tumor macrophage populations could impair wound healing or alter responses to infection even when conventional acute toxicity appears low [19,20,21,22,23]. Such risks strengthen the rationale for spatially restricted delivery and for evaluating off-tumor macrophage function rather than relying only on standard organ-toxicity measurements. The evidence summarized in Table 3 is used here to separate formulation performance from macrophage-specific uptake/exposure, molecular reprogramming, and downstream immune effects. To apply these criteria to the existing literature rather than only prospectively, the principal studies summarized in Table 1 were re-extracted for particle size/PDI, Cur loading or encapsulation, administered Cur dose, route, release conditions, tumor model, and the type and resolution of exposure measurement. Supplementary Table S1 distinguishes carrier localization from parent-drug measurement, total or formulated drug from released drug, and whole-tumor from cell-resolved exposure; NR is used only for variables confirmed as not measured or not reported after review of the full primary article and available Supplementary Material.

## 7. Translational Challenges and Future Molecular Engineering Directions

Translation will require simultaneous control of three forms of heterogeneity: nanoparticle behavior, tumor architecture, and macrophage state. Nanoparticles acquire a biomolecular corona that can mask targeting ligands or create new biological identities [88,89]. Only a fraction of the injected dose reaches many solid tumors [83], and tumor entry, penetration, and retention depend on vascular and stromal properties [84,85,86,87,88,89,90,91,92]. TAM-targeting adds another layer because the same phagocytic biology that favors uptake can divert particles to the liver and spleen. Curcumin adds a final chemical constraint: instability and extensive metabolism mean that carrier integrity and release must be linked to the measurement of the active parent compound, not only to fluorescent labels.

Human TAM heterogeneity is an equally important barrier. Single-cell and transcriptional studies show macrophage programs that are tumor-specific and not faithfully represented by a universal M2 target [24,25,26,27]. Future delivery should therefore move from phenotype-targeted nanomedicine toward molecular-state-targeted nanomedicine. Instead of asking whether a carrier binds ‘M2 macrophages’, investigators should define the suppressive macrophage state they seek to modulate—for example, a CSF1R-dependent, SPP1-high, hypoxic, TREM2-associated, or STAT3/IL-10-dominant state—and validate the target in patient tissue before engineering the ligand. This approach also makes biomarkers possible.

Single-cell RNA sequencing, spatial transcriptomics, multiplex imaging, and proteomic/phosphoproteomic methods can define state and location, but they should be integrated with pharmacology. One priority is cell-resolved PK/PD: isotope-labeled or analytically traceable curcumin measured in sorted TAMs, tumor cells, dendritic cells, and off-target macrophages, followed by pathway assays in those same populations. A second priority is spatial PK/PD: mapping carrier and drug distribution relative to hypoxic niches, SPP1-positive macrophages, T-cell-excluded regions, and tumor vasculature. A third is functional perturbation: macrophage depletion, receptor blockade, or pathway rescue to determine whether the therapeutic effect truly depends on TAM reprogramming.

Relevant baseline variables may include TAM abundance and state [24,25,26,27], PD-L1 distribution and IFN/hypoxia signaling [74,113], T-cell exclusion and TGF-β activity [37,62,63,108,109], and myeloid PI3Kγ or TREM2 programs [35,36,115,116]. A curcumin nanotherapy is unlikely to sensitize every checkpoint-resistant tumor through the same route. Therapeutic resistance in the same tumors may also be reinforced by cancer stem-cell plasticity sustained by epigenetic programs; recent evidence-weighted synthesis indicates that curcumin can modulate several such programs, although this evidence is mechanistically distinct from TAM reprogramming and does not establish CSC eradication [117]. The most rational clinical concept would select tumors in which the targeted macrophage circuit is demonstrably active and then use a pharmacodynamic biomarker to confirm modulation. This is a more credible translational path than treating nanocurcumin as a universal immunotherapy adjuvant.

Manufacturing and reporting standards are equally important. Biomimetic membranes and engineered vesicles require rigorous control of source cells, membrane composition, residual nucleic acids/proteins, batch consistency, sterility, and storage stability. Conventional polymeric or albumin carriers require reproducible size, loading, release, and ligand density. Minimum information standards for bio-nano experiments [118] should be applied alongside immunological standards that define macrophage source, polarization conditions, marker panels, gating, and functional assays. Industry experience shows that complexity that improves preclinical performance can simultaneously make scale-up and regulatory characterization harder [119,120]. The optimal platform may therefore be the simplest architecture that achieves verified cell-specific delivery and molecular effect.

A practical future research program can be organized into five linked experiments. First, identify a human TAM state associated with checkpoint resistance using single-cell/spatial data. Second, validate a surface receptor or microenvironmental feature that can be targeted without excessive off-tumor macrophage binding. Third, engineer a curcumin carrier and quantify cell-resolved exposure. Fourth, demonstrate causal pathway engagement and macrophage function in patient-derived or humanized systems. Fifth, test combination therapy with prospectively defined checkpoint and immune endpoints. Completion of this chain would move the field from promising formulation biology to a mechanistically credible immunopharmacology. Figure 6 integrates a prospective translational workflow with the independent D1–D8 evidence domains, linking human TAM-state selection and precision delivery to cell-resolved exposure, mechanistic validation, immune/checkpoint testing, and clinical translation.

## 8. Conclusions

Curcumin nanodelivery, TAM reprogramming, and checkpoint-directed immunotherapy form a scientifically coherent but still emerging research axis. Current nanoplatform studies support improved curcumin delivery and, in selected systems, TAM-associated uptake/remodeling or broader immune-microenvironment modulation [40,42,43,45]. Independent macrophage-focused studies further support Cur-associated regulation of STAT6 and PI3K-AKT/STAT3 signaling, including functional effects in patient-derived TAMs [64,67,68]. Checkpoint-related studies provide evidence of therapeutic benefit in selected combination settings and PD-L1-associated models [44,100,101], while ICD-oriented platforms provide complementary evidence for downstream immune activation [45,48,93]. However, these components are generally demonstrated in different experimental systems. No single study currently establishes the complete sequence from cell-resolved parent-Cur exposure through causal TAM reprogramming to enhanced checkpoint response. The strength of the current literature therefore lies in convergent evidence across complementary mechanistic domains rather than in demonstration of a complete causal chain.

The decisive advance will come from linking delivery precision to molecular causality. Future studies should distinguish tumor targeting from TAM targeting, measure released curcumin in relevant cell populations, validate pathway engagement in those cells, demonstrate macrophage function beyond M1/M2 markers, and test whether that function is necessary for checkpoint benefit. Human TAM heterogeneity, protein-corona effects, mononuclear-phagocyte clearance, manufacturing complexity, and dose-dependent curcumin pharmacology must be incorporated early rather than treated as late translational details. The D1–D8 framework provides a structured way to assess these requirements independently, without allowing efficacy in one domain to substitute for missing evidence in another. Progress toward clinically meaningful Cur immunonanomedicine will depend on experimentally connecting these domains while preserving the distinction between demonstrated mechanism and biological plausibility.

## Figures and Tables

**Table 1:** Comparative appraisal of principal experimental studies relevant to curcumin nanodelivery, TAM remodeling, and immune/checkpoint outcomes.

Study	Delivery/Model	Key Experimental Design	Representative Quantitative Outcome	Critical Interpretation
[40]	M2pep-Cs-Cur NPs; 4T1 TNBC	NPs 10 mg/kg IV q2d; anti-PD-L1 12.5 mg/kg IP q3d; n = 6; Control/NP/NP+anti-PD-L1	~50% survival enhancement reported with the NP + anti-PD-L1 regimen	TAM uptake/reprogramming supported; no anti-PD-L1-only arm or macrophage-dependence test, so combination benefit ≠ formal sensitization/synergy/TAM necessity
[41]	Cur-PDA@CM; CT26 CRC	Cur-equivalent dose 5 mg/kg IV; n = 6/group; PBS/Cur/Cur-PDA/Cur-PDA@CM/Cur-PDA@CM+NIR	M1-TAM proportion ↑21%; ↑28% with NIR	TAM polarization measured, but macrophage-membrane coating demonstrates tumor homing, not TAM-specific targeting; TAM necessity untested
[42]	Cur-CSNP + PDT; 4T1 TNBC	Cur or Cur-CSNPs (Cur 2.5 mg/kg) intratumorally on d0 and d3; 980 nm, 1 W/cm^2^, 20 min after 4 h; bilateral 4T1/BALB/c; n = 5 for tumor-growth efficacy	Primary tumors eradicated in 3/5 mice; BMDM CD86^+^ 17.2% → 33.1% ↑; CD206^+^ 73.6% → 35.8% ↓; distal CD4^+^/CD8^+^ infiltration ↑	Supports TAM remodeling during Cur-PDT, but Cur, PDT, and ICD contributions are intertwined; TAM dependence not demonstrated
[43]	Dual pH-sensitive Cur/anti-PD-1 nanodrug; B16F10	Cur 2 mg/kg + anti-PD-1 1.2 mg/kg on d6/9/12/15; n = 5; free and single-component controls	Tumor Cur > 10 μM for 12–48 h; combination gave strongest immune/tumor response	Strong component-level checkpoint controls; macrophage NF-κB modulation supported, but TAM necessity was not tested.
[44]	Cur/anti-PD-L1 inflammation-responsive nanodrug; HCC	Hepa1-6/C57BL/6; PBS/Cur@CPP/aPD-L1@CPP/Cur@CPP-aPD-L1; Cur 4 mg/kg + aPD-L1 2 mg/kg IV every 3 d (d0–30); n = 5 for efficacy	CD3^+^/CD8^+^ T cells ↑; F4/80^+^/CD206^+^ M2 macrophages ↓; PD-L1^+^ cells/Tregs ↓; MCP-1/COX-2/TGF-β ↓, IFN-γ ↑; dual nanodrug showed strongest tumor suppression	Integrated Cur + checkpoint co-delivery with immune remodeling; uptake evidence is carrier/surrogate based rather than direct intracellular parent-Cur measurement, and TAM necessity was not established.
[45]	PD1@Cur-PLGA; 4T1	Cur 4 mg/kg IV d0/2/4/6; n = 5; PBS/Cur/PD1-NV/Cur-PLGA/PD1@Cur-PLGA	Mature DCs 1.77× and CD8^+^ T cells 1.60× PBS	Strong ICD/DC/CD8 evidence with checkpoint-binding carrier; TAM mechanism not central or demonstrated.
[46]	Cu_9_S_8_@PCM@Cur + PTT; AKR/C57	n = 6; Control/anti-PD-1/nanocomposite+laser/combination; efficacy dose not explicitly stated	Combo tumor weight 18.81× below anti-PD-1 and 5.52× below nano+laser; CD8 infiltration 1.9× control	Clear combination efficacy, but effect integrates PTT, Cur, ICD, and checkpoint blockade; not evidence of TAM-dependent sensitization.
[47]	Anti-PD-L1-PBA-Cur ADC; HCT116 colon cancer	Anti-PD-L1 (10 mg/kg), free Cur (50 mg/kg), anti-PD-L1 + Cur (10 + 50 mg/kg), or anti-PD-L1-PBA-Cur ADC (nominal 10 mg/kg); q3d ×4 wk; administration route and ADC dose-normalization basis NR; n NR	ADC IC50 0.520 vs. Cur 5.504 μg/mL	Tumor-cell PD-L1/CSN5 and PBMC activation studied; no TAM evidence; nude-mouse ADC study, not a conventional checkpoint-combination experiment.
[48]	Cur/miR-16-5p NBs + ultrasound; HCC	n = 5/group; Control/NBs+US/Cur+US/Cur-NBs+US/miR-16-NBs+US/Cur/miR-16-NBs+US; 200 μL IV treatment, US 1 MHz, 1.5 W/cm^2^, 120 s; every other day	Tumor-volume inhibition 53.39%; mass inhibition 50.71%	Cur drives ICD while miR-16-5p suppresses PD-L1; no checkpoint antibody and no TAM-specific mechanism; allogeneic tumor/host model is an added limitation.
[93]	CMCS Cur+DOX NPs; 4T1 breast cancer	n = 5; 7 groups; tail vein q1d × 7; numeric Cur/DOX doses not reported (NR)	Tumor inhibition 84.27±1.72%; IFN-γ 1891.72±158.01 pg/mL	Cur+DOX-associated ICD markers and supportive downstream immune responses; no direct functional immune-restoration assay, TAM-specific mechanism, or checkpoint-therapy evidence
[50]	Dendrosomal Cur; 4T1/BALB/c	40 or 80 mg/kg for 35 d; n = 9/group; saline control	Final tumor volume 1.11 → 0.31/0.27 cm^3^; IL-12 5.41× and STAT4 21.65× at 80 mg/kg	Supports macrophage-associated M1/M2 transcriptional remodeling; lacks cell-resolved functional/depletion evidence establishing macrophage causality
[51]	Cur-CD68Ab or Cur phytosome; GL261 GBM	Vehicle n = 10; CCP phytosome 2 mg formulation/mouse IP q72h for 1 month (n = 10); CC-CD68Ab adduct 120 pmol/mouse IN (Cur:Ab 1:1), q72h × 4 then q7d × 4 (n = 8); treatment from d10 after GL261 implantation	50% tumor remission; day-90 survival: 70% adduct, 60% CCP, 0% control	Iba1+ TAM/microglial ARG1↓/iNOS↑; however, CD68Ab also targets CD68-high GBM cells, so TAM-specific therapeutic causality is unresolved.
[52]	Cur phytosome; GL261/C57BL/6	CCP 2 mg/mouse IP daily × 5; NK1.1-depletion and IL-12-blockade cohorts	M1-type macrophages ≈50–60% of TAMs; NK depletion partially reversed effects	Strong macrophage-state and NK-dependence evidence; does not prove that macrophages themselves are necessary for tumor control.
[53]	Liposomal TriCurin; GL261 GBM	Vehicle/Cur-liposome/TriCurin-liposome; long-term n = 4/group	GBM remission 50% with TriCurin vs. 25% with Cur-liposome	Clear TAM/microglial repolarization, but Cur-only attribution is impossible because TriCurin contains Cur, epicatechin gallate, and resveratrol.

Note: ADC, antibody–drug conjugate; CD68Ab, anti-CD68 antibody; CM, macrophage cell membrane; CMCS, carboxymethyl chitosan; CCP, curcumin phytosome; CRC, colorectal cancer; Cs, chitosan; CSN5, COP9 signalosome subunit 5; Cur, curcumin; DC, dendritic cell; DOX, doxorubicin; GBM, glioblastoma; HCC, hepatocellular carcinoma; ICD, immunogenic cell death; IFN-γ, interferon-γ; IL-12, interleukin-12; IN, intranasal; IP, intraperitoneal; IV, intravenous; M1/M2, conventionally defined macrophage polarization phenotypes; M2pep, M2 macrophage-targeting peptide; miR-16-5p, microRNA-16-5p; NB, nanobubble; NIR, near-infrared; NF-κB, nuclear factor kappa B; NP, nanoparticle; NV, nanovesicle; PBA, phenylboronic acid-based linker; PBMC, peripheral blood mononuclear cell; PCM, phase-change material; PD-1, programmed cell death protein 1; PD-L1, programmed death-ligand 1; PDA, polydopamine; PDT, photodynamic therapy; PLGA, poly(lactic-co-glycolic acid); PTT, photothermal therapy; STAT4, signal transducer and activator of transcription 4; TAM, tumor-associated macrophage; TME, tumor microenvironment; TNBC, triple-negative breast cancer; US, ultrasound.

**Table 2:** TAM-targeted nanomedicine precedents that provide transferable engineering lessons for curcumin.

Engineering Precedent	Targeting Principle	Cargo/Pathway	Transferable Lesson for Curcumin	Key Caveat
M2pep proapoptotic peptide [75]	M2pep binding to murine M2-like TAMs	TAM depletion	A macrophage-selective ligand can discriminate the target from resident/off-target immune cells	Human-state specificity needs validation
M2pep-PLGA-PLX3397 [76]	M2pep surface functionalization	CSF1R inhibition	Compare targeted vs. untargeted carrier and free cargo	CD206-rich mouse model may not reflect all human TAMs
Dual M2pep/SR-B1 siRNA NP [77]	Two-receptor targeting	CSF1R siRNA	Cell selectivity can be paired with molecular target knockdown	Complexity may increase the manufacturing burden
CDNP-R848 [78]	Phagocyte-favoring cyclodextrin NP	TLR7/8 activation	Measure TAM function and checkpoint synergy, not markers alone	Intratumoral/systemic translation differs
M2pep-CSF1R siRNA [79]	Peptide-targeted cyclodextrin	CSF1R silencing	Link uptake → knockdown → TAM function → T-cell effect	Gene cargo differs from small-molecule Cur
Mannosylated R848 NP [80]	CD206/mannose receptor-mediated uptake	TLR7/8 activation	Dual targeting of TAMs and DCs may create coordinated innate immunity	CD206 is not TAM-exclusive
Mannosylated albumin NP [81]	Tumor + M2 macrophage dual targeting	Drug-resistant tumor therapy	Albumin/ligand architecture can combine tumor and myeloid recognition	Need to distinguish tumor-cell from macrophage contribution

Note: These are mechanistically supportive engineering precedents, not direct evidence that curcumin behaves identically in the same platforms.

**Table 3:** Multidimensional evidence framework for critical appraisal of curcumin immunonanomedicine studies.

Domain	Evidence Question	Direct Evidence	Indirect/Supportive Evidence	What Should Not Be Inferred
D1 Pharmaceutical/formulation performance	Is the pharmaceutical system adequately characterized?	Size/PDI, loading, stability, release under relevant conditions	Limited physicochemical characterization	Tumor or TAM delivery
D2 Tumor delivery/exposure	Is parent-curcumin exposure in the tumor quantitatively demonstrated?	Quantitative measurement of parent Cur in tumor tissue or tumor-resolved parent-Cur PK/biodistribution using an analytical drug assay	Carrier or surrogate-dye imaging/localization without quantitative parent-Cur measurement	TAM-specific exposure
D3 TAM-specific uptake and intracellular parent curcumin exposure	Does the intended macrophage population take up the carrier/drug and receive pharmacologically relevant Cur?	Cell-resolved uptake plus quantitative parent-Cur measurement where available	Colocalization or carrier fluorescence alone	Functional TAM reprogramming
D4 Cell-specific pathway modulation	Is pathway modulation directly measured in the intended cell?	Cell-specific pathway activity/readouts, including phosphorylation, expression, nuclear translocation, or pathway-linked transcription measured after treatment	Whole-tumor or non-cell-resolved pathway changes; downstream-marker inference; docking or network predictions	Causal pathway dependence, direct Cur–protein binding/occupancy, or macrophage functional change
D5 Functional TAM reprogramming	Does treatment alter macrophage function?	Phagocytosis, antigen presentation, cytokine/chemokine function, suppression, tumor-support assay	Phenotypic markers alone	Adaptive immune restoration
D6 Downstream immune restoration	Are other immune compartments functionally improved?	Functional DC/NK/T-cell assays, cytotoxicity, antigen-specific responses	Cell-number or infiltration changes alone	Checkpoint sensitization
D7 Checkpoint-therapy response	Does the Cur platform alter response to PD-1/PD-L1-directed treatment?	Appropriate factorial comparison including checkpoint monotherapy; synergy/resistance endpoints where claimed	Combination benefit without adequate monotherapy comparison	TAM dependence or formal synergy
D8 Translational relevance	Is there evidence supporting human translation?	Human TAMs/patient material, humanized models, PK/PD, safety, manufacturability, biomarkers	Conventional murine/in vitro models	Clinical efficacy

Note: The domains are independent rather than cumulative. In D1–D8, “D” denotes domain. For study-level appraisal, each domain is classified as directly demonstrated (D), indirect/supportive (I), or not measured/reported (NR). Evidence in one domain is not used to infer evidence in another. Within D4, a D code denotes directly measured cell-specific pharmacodynamic pathway modulation. D4 = D does not by itself establish causal pathway dependence; causal attribution requires an appropriate perturbation, inhibition, knockdown, overexpression, or rescue experiment. Direct molecular target engagement requires separate evidence of drug–target binding or occupancy.

## Data Availability

No new data were created or analyzed in this study. The evidence synthesized in this review is available in the cited publications and Supplementary Materials.

## Supplementary Materials

The following supporting information can be downloaded at: https://www.mdpi.com/article/10.3390/pharmaceutics18101295/s1, Table S1: Detailed evidence matrix for principal experimental studies relevant to curcumin nanodelivery, TAM remodeling, and immune/checkpoint outcomes. Panel A. Pharmaceutical, model, exposure, and comparator characteristics. Panel B. Mechanistic, immune, safety, limitation, and D1–D8 evidence coding; Table S2: Chemical identity and evidentiary classification of curcumin-related compounds and formulations discussed in the review.

## Abbreviations

The following abbreviations are used in this manuscript:

ARG1	Arginase 1
BSA	Bovine serum albumin
CAF	Cancer-associated fibroblast
CCL2	C-C motif chemokine ligand 2
CCR2	C-C chemokine receptor 2
CRC	Colorectal cancer
CSF1	Colony-stimulating factor 1
CSF1R	Colony-stimulating factor 1 receptor
Cur	Curcumin
DC	Dendritic cell
DOX	Doxorubicin
GBM	Glioblastoma
GSDME	Gasdermin E
HCC	Hepatocellular carcinoma
HIF-1α	Hypoxia-inducible factor 1 alpha
ICD	Immunogenic cell death
IFN-γ	Interferon gamma
iNOS	Inducible nitric oxide synthase
LC-MS	Liquid chromatography–mass spectrometry
LUAD	Lung adenocarcinoma
MAPK	Mitogen-activated protein kinase
MDSC	Myeloid-derived suppressor cell
miR	MicroRNA
NF-κB	Nuclear factor kappa B
NK	Natural killer
NP	Nanoparticle
PD	Pharmacodynamics
PDA	Polydopamine
PK/PD	Pharmacokinetics/pharmacodynamics
PLGA	Poly(lactic-co-glycolic acid)
ROS	Reactive oxygen species
siRNA	Small interfering RNA
STAT	Signal transducer and activator of transcription
TAM	Tumor-associated macrophage
TCR	T-cell receptor
TGF-β	Transforming growth factor beta
TIDE	Tumor Immune Dysfunction and Exclusion
TME	Tumor microenvironment
TNBC	Triple-negative breast cancer
TNF-α	Tumor necrosis factor alpha
VEGF	Vascular endothelial growth factor
