# Supplementary material for: Curcumin Nanodelivery for Tumor-Associated Macrophage Reprogramming and Immune-Checkpoint Sensitization: Engineering the Tumor Immune Microenvironment
Source: Pharmaceutics. 2026 Oct 13;18(10):1295. doi: 10.3390/pharmaceutics18101295 (PMC13708596; doi:10.3390/pharmaceutics18101295)
Supplement: Supplementary file 1 [file pharmaceutics-18-01295-s001.zip › pharmaceutics-4575816-supplementary.pdf]

Supplementary Information

Curcumin Nanodelivery for Tumor-Associated Macrophage Reprogramming and Immune-Checkpoint Sensitization: Engineering the Tumor Immune Microenvironment

Supplementary Table S1. Detailed evidence matrix for principal experimental studies relevant to curcumin nanodelivery, TAM remodeling, and immune/ checkpoint outcomes. **Panel A.** Pharmaceutical, model, exposure, and comparator characteristics

Refs.	System / cargo	Size / PDI	Cur loading / EE	Cur dose, route, schedule	Release behavior	Tumor model / host / n	Macrophage source / readout	Exposure measurement	Intracellular parent Cur in TAMs?	Key controls / comparators
**Dong et al. [40]**	M2pep-chitosan-Cur nanoparticles; M2pep intended for M2-like macrophage targeting	182 ± 15.3 nm; PDI NR	EE ~84%; loading 5.6%	M2pep-Cs-Cur NPs 10 mg/kg IV q2d (formulation dose; parent-Cur-equivalent dose not explicitly reported); anti-PD-L1 12.5 mg/kg IP q3d	First 2 h: ~20% (pH 7.4), ~30% (6.5), ~40% (5.0)	4T1 TNBC; animal-method description internally inconsistent (C57BL/6J stated in one section; female nude mice described for implantation); efficacy n=6	THP-1-derived macrophages in vitro; tumor macrophage uptake/polarization analyses in vivo	Fluorescence imaging/flow cytometry for nanoparticle uptake; tumor-level efficacy	No. Uptake signal did not quantify released parent Cur inside TAMs	Control, NP, NP + anti-PD-L1. No anti-PD-L1-only arm; no macrophage depletion/blockade/ rescue
**Liu et al. [41]**	Self-assembled Cur-PDA coated with J774A.1 macrophage membrane (Cur-PDA@CM); tumor-homing biomimetic carrier	Final ~180 nm; exact final PDI NR (within acceptable range)	Cur loading 16.8 ± 1.2% (HPLC)	Cur-equivalent dose 5 mg/kg IV across Cur-containing groups; NIR 808 nm, 1 W/cm², 5 min at 24 h post-injection	pH 7.4, 37°C; ~50% cumulative Cur release from final system at 72 h; NIR accelerates release	CT26 in female BALB/c; efficacy n=6/group	Tumor FACS: CD45/F4/80 with CD86/CD206	DiR-labeled carrier tumor imaging; tumor phenotype analysis	No	PBS, free Cur, Cur-PDA, Cur-PDA@CM, Cur-PDA@CM + NIR. Macrophage membrane coating is not evidence of TAM-directed delivery
**Zhang et al. [42]**	Cur-loaded DMSN/UCNP core-satellite particles (Cur-CSNPs); Cur acts as photosensitizer + HIF-1α modulator	~142.2 nm; PDI NR; zeta +18.2 mV	EE 18.66%; DL 2.05%	Cur 2.5 mg/kg intratumoral into primary 4T1 tumor; 980 nm, 1 W/cm², 20 min; repeated once after 3 d	ROS/NIR-triggered; markedly faster after 980-nm irradiation	Bilateral orthotopic 4T1 in female BALB/c; efficacy n=5; selected tissue/IHC n=3	C57BL/6-derived BMDMs co-cultured with treated 4T1 cells; distal-tumor CD86/CD206 IHC	Local intratumoral administration; tumor-cell Cur fluorescence/lysosomal localization; no quantitative in vivo parent-Cur PK	No. No direct Cur quantification in BMDMs/TAMs	Control, free Cur, Cur-CSNPs, Cur-CSNPs + 980 nm; untreated distal tumor models abscopal response
**Xiao et al. [43]**	Dual pH-sensitive CUR@PPC-aPD-1 nanodrug; Cur + anti-PD-1	~50 nm at pH 7.4; ~43 nm at pH 6.5	NR in main summary used here	Cur 2 mg/kg + aPD-1 1.2 mg/kg IV on days 6, 9, 12, 15	~70% aPD-1 release by 15 h at pH 6.5; Cur 12.7% at 48 h pH 7.4; faster under lysosome-like acidity	B16F10 in C57BL/6; n=5/group for efficacy	RAW264.7 macrophages; tumor immune analyses	LC-MS quantified whole-tumor Cur >10 μM from 12–48 h; tracer/MRI carrier localization	No. Whole-tumor parent Cur measured, not TAM intracellular Cur	PBS, free Cur formulation, free aPD-1, Cur-only nanocarrier, antibody-bearing carrier, dual nanodrug
**Wang et al. [44]**	pH-sensitive Cur@CPP-aPD-L1 micelle; Cur + anti-PD-L1	101.7 nm; PDI 0.121 (blank 87.2 nm; PDI 0.111)	Cur 6.1% loading; aPD-L1 5% loading	Cur 4 mg/kg + aPD-L1 2 mg/kg IV q3d, day 0–30	pH-responsive: Cur released preferentially at pH 5.5; aPD-L1 released preferentially at pH 6.5	Hepa1-6 in C57BL/6, immunocompetent; efficacy n=5; immune assays n=3	RAW264.7 in vitro; tumor F4/80 and F4/80+CD206 in vivo	Nile-red/DiR surrogate uptake and biodistribution; Alexa488-aPD-L1 binding; no quantitative parent-Cur tissue assay	No	PBS, Cur@CPP, aPD-L1@CPP, Cur@CPP-aPD-L1; component-level checkpoint controls
**Liao et al. [45]**	PD1-overexpressing tumor-cell	Cur-PLGA 149.4 ± 1.5 nm; final	Cur loading quantified by UV-vis; exact	Cur-equivalent 4 mg/kg IV on days 0, 2, 4, 6	Cur release: Cur-PLGA 59.638% and PD1@Cur-	4T1/BALB/c (n=5 efficacy); RM-1/C57BL/6 validation	No TAM-specific assay	DiR-labeled nanoparticle biodistribution; tumor	No	PBS, free Cur, PD1 nanovesicles, Cur-PLGA, PD1@Cur-PLGA

	nanovesicle-coated Cur-PLGA (PD1@Cur-PLGA); engineered PD1 provides checkpoint binding	177 ± 5.7 nm	DLE/DLC not reported in extracted main-text values		PLGA 58.315% at 84 h			targeting; carrier-associated signal		
**Ji et al. [46]**	Hollow Cu9S8@PCM@Cur; thermally triggered Cur release; anti-PD-1 added in checkpoint experiment	161.8 ± 1.9 nm; PDI 0.330	Cur loading efficiency 11.7%	Exact Cur-equivalent dose for efficacy/checkpoint regimen not independently stated in main efficacy section; IV nanoparticle studies used 2.5 mg/kg; laser 980 nm, 1 W/cm²	30 min: 10.03% at 37°C vs 54.1% at 44°C; NIR/power-dependent	AKR subcutaneous tumor in male C57 mice; efficacy and checkpoint studies, n=6	No TAM-specific assay	DiD-labeled nanoparticle tumor accumulation; tumor-cell PD-L1 and immune readouts	No	Base 7-arm PTT/Cur controls; checkpoint study includes control, anti-PD-1, nano+laser, nano+laser+anti-PD-1
**Ding et al. [47]**	ROS-responsive anti-PD-L1-PBA-Cur antibody-drug conjugate (ADC)	NR	Conjugate composition reported; conventional EE/DL not applicable/NR	HCT116 nude mice: anti-PD-L1 10 mg/kg; free Cur 50 mg/kg; free combination at 10 + 50 mg/kg; anti-PD-L1-PBA-Cur ADC nominally 10 mg/kg; all q3d for 4 wk. ADC dose basis (total conjugate, antibody-equivalent, or Cur-equivalent) and administration route not specified.	H2O2/ROS-responsive Cur release from PBA linkage	HCT116 xenograft in nude mice; n not stated in accessible methods	No TAM assay	Tumor response; PD-L1/CSN5; no parent-Cur cell-resolved PK	No	PBS, anti-PD-L1, Cur, anti-PD-L1 + Cur, ADC
**Xu et al. [48]**	Ultrasound-responsive Cur/miR-16-5p co-loaded nanobubbles; UTMD	546.2 ± 8.5 nm; PDI NR	Cur EE ~55.6%	200 μL IV treatment via tail vein, followed by US (1 MHz, 1.5 W/cm², 50% duty cycle, 120 s) for all non-control groups; treatments every other day; in vivo Cur mg/kg NR	Ultrasound-triggered nanobubble rupture/release; no conventional cumulative release value extracted	Hepa1-6 subcutaneous in BALB/c (allogeneic strain context); n=5/group	No TAM assay	Formulation/cellular uptake and tumor response; Cur concentration inferred from EE in vitro, not cell-resolved in vivo	No	Control, NBs+US, Cur+US, Cur-NBs+US, miR-16-NBs+US, Cur/miR-16-NBs+US
**Shiri et al. [50]**	Dendrosomal curcumin (DNC)	NR	NR	40 or 80 mg/kg; daily for 35 d; administration route not clearly reported in accessible primary article	NR	4T1 metastatic breast cancer in BALB/c; 27 mice equally divided (n=9/group)	Macrophage presence in tumor/spleen; tissue STAT4/IL-12 vs STAT3/IL-10/Arg1 gene expression	No pharmacokinetic or cell-resolved Cur exposure measurement	No	Control, DNC 40 mg/kg, DNC 80 mg/kg
**Mukherjee et al. [51]**	Curcumin-CD68 antibody adduct	NR	Not reported as	CC-CD68Ab adduct: 120	NR	GL261 syngeneic glioblastoma; survival	Tumor-associated Iba1+ microglia/macrophages;	Targeted adduct delivery and systemic phytosome;	No	Vehicle vs CCP vs CC-CD68Ab; important limitation:

	(intranasal) and commercial curcumin phytosome (CCP/Meriva; i.p.)		conventional nanoparticle EE/DL	pmol/mouse per IN administration (Cur:Ab 1:1), q72h ×4 then q7d ×4; CCP phytosome: 2 mg formulation/mouse IP q72h for 1 month. Both treatments started d10 after GL261 implantation; CCP mass is not pure-Cur mass.		cohorts vehicle n=10, CCP n=10, CC-CD68Ab n=8	ARG1/iNOS phenotype and NF-κB/p65–STAT1 signaling readouts; no independent macrophage functional assay	no quantitative intracellular parent Cur in TAMs		CD68 also expressed by GL261 tumor cells
**Mukherjee et al. [52]**	Phytosomal curcumin (CCP/Meriva)	NR	Commercial phytosomal curcuminoid preparation; conventional EE/DL NR	CCP 2 mg/mouse IP daily ×5 (short-term mechanistic study)	NR	GL261/C57BL/6 immunocompetent; vehicle n=5, CCP n=5, CCP+NK1.1 n=4 in key experiment	Iba1+ TAMs; ARG1/iNOS, IL-10/IL-12, STAT1/STAT3; peripheral macrophage recruitment	Systemic CCP produced sub-IC50 plasma Cur in prior/linked PK; no TAM intracellular parent-Cur quantification	No	Vehicle, CCP, CCP+NK1.1; NK1.1 antibody 100 µg/mouse; separate IL-12 blockade experiments
**Mukherjee et al. [53]**	Liposomal TriCurin (TrLp): Cur + epicatechin gallate + resveratrol at C:E:R 4:1:12.5	Liposomal particles; median hydrodynamic diameter ~200 nm for TrLp (~260 nm for Cur-liposome comparator)	Multi-agent liposomal formulation; Cur-specific EE/DL not used for attribution	TrLp 1.28 mM+ (200 µL/mouse, containing 0.256 µmol Cur) i.p.; short-term q24 h ×5 from day 12; long-term q72 h for 60 days. Cur-liposome comparator: 5 mM, same volume/schedule	NR	GL261 glioblastoma in male C57BL/6 mice; n=3/group short-term mechanistic cohort and n=4/group long-term survival cohort	Tumor-associated microglia/macrophages; M2-like→M1-like changes; activated NK recruitment	Plasma parent Cur: peak ~0.61 µM at 10 min and ~210 nM maintained at 60 min after TrLp; no TAM intracellular parent-Cur measurement	No	Vehicle, Cur-liposome, TrLp; multi-agent attribution limits Cur-specific inference
**Wu et al. [93]**	CMCS-coated pH-responsive Cur + doxorubicin nanoparticles (CMCS-D+C/NPs)	Final CMCS-D+C/NPs: 191.87 ± 5.24 nm; uncoated D+C/NPs: 163.92 ± 4.02 nm; PDI NR	Cur EE 64.39 ± 3.45%; DOX EE 50.99 ± 4.15% for final CMCS-D+C/NPs	Tail vein q1d ×7; numeric Cur/DOX doses NR	pH-responsive release	4T1 mouse tumor; 7 groups, n=5/group	No TAM assay	Ce6 surrogate tumor-accumulation imaging; no parent-Cur cell-resolved measurement	No	PBS, free DOX, free Cur+DOX, CMCS-C/NPs, CMCS-D/NPs, D+C/NPs, CMCS-D+C/NPs

Panel A note: NR = not measured/reported after verification of the full primary article and available supplementary material; inability to access or verify a source is not coded as NR. Carrier-associated fluorescence, surrogate dyes, and whole-tumor measurements are explicitly distinguished from released parent-Cur measurement. Doses refer to the principal in vivo efficacy design unless otherwise stated. Reference numbers correspond to the main manuscript.

Supplementary Table S1 (continued) Panel B. Mechanistic, immune, safety, limitation, and D1–D8 evidence coding

Refs.	Pathway / functional evidence	TAM evidence	Downstream immune evidence	Checkpoint design / response	Synergy / resistance evidence	Dependence test	Safety	Principal limitation	D1	D2	D3	D4	D5	D6	D7	D8
**Dong et al. [40]**	COX-2 downregulated; COX-2 overexpression attenuated NP-induced macrophage reprogramming and	Targeted macrophage uptake/reprogramming; TNBC co-culture effects partly	Immune/checkpoint-associated tumor benefit; details less causally resolved than uptake/state evidence	NP + anti-PD-L1 outperformed NP alone, but anti-PD-L1 monotherapy absent	Combination benefit reported; formal synergy/resistance reversal not established	No macrophage depletion/blockade/rescue	Reported tolerability; conventional safety only	Animal-host inconsistency; no anti-PD-L1-only arm; no macrophage	D	I	I	D	D	I	I	NR

	restored TNBC proliferation/migration/invasion; IL-17/TNF-α changes supportive	reversed by COX-2 overexpression; no intracellular parent-Cur assay						necessity; no TAM parent-Cur PK								
**Liu et al. [41]**	ROS/photothermal + Cur effects; no macrophage-specific causal pathway test	Tumor CD86/CD206 shifts after therapy; macrophage membrane is tumor-homing camouflage, not TAM targeting	No strong adaptive immune functional endpoint	No checkpoint therapy	NIR adds multimodal benefit; no formal checkpoint synergy	No	Body weight/organ histology and serum safety assessments	TAM phenotype based mainly on marker proportions; carrier homing does not establish TAM delivery	D	I	NR	I	I	NR	NR	NR
**Zhang et al. [42]**	HIF-1α inhibited in tumor cells after Cur-CSNP PDT; no rescue/perturbation proving HIF-1α necessity for TAM shift	BMDM CD86 17.2→33.1%; CD206 73.6→35.8%; distal tumor CD86↑/CD206↓	CD4+/CD8+ infiltration increased; abscopal growth suppression	No checkpoint inhibitor	PDT + Cur multifunctionality; not checkpoint synergy	No macrophage depletion/rescue	Stable body weight; major-organ H&E without toxic damage	Macrophage “repolarization” primarily marker-based; Cur delivered to tumor cells, not quantified in TAMs	D	I	NR	D	I	I	NR	NR
**Xiao et al. [43]**	CUR@PPC suppresses NF-κB/pho-p65, CCL22, TGF-β, IL-10 and PD-L1 in RAW264.7/tumor cells	Macrophage pathway/cytokine modulation; nanodrug uptake inferred/visualized, no TAM parent-Cur quantification	Reduced suppressive cytokines/Tregs; enhanced tumor T-cell infiltration/function	Actual anti-PD-1 included; dual nanodrug compared with single components	Strong component-level combination design; macrophage necessity not tested	No macrophage depletion/block/rescue	Major-organ/biodistribution assessments reported	Whole-tumor Cur LC-MS does not establish Cur concentration inside TAMs	D	D	I	D	D	D	D	NR
**Wang et al. [44]**	NF-κB/IKBα pathway suppressed; macrophage/tumor MCP-1, TGF-β, COX-2/PD-L1 reduced	RAW264.7 functional cytokine/transcript changes + fewer F4/80+ and F4/80+CD206+ tumor cells	CD3/CD8↑, Treg↓, IFN-γ↑; immune remodeling	Cur@CPP-aPD-L1 compared with Cur@CPP and aPD-L1@CPP; actual aPD-L1 therapy	Combination superior to single-component carriers; formal mathematical synergy not reported	No macrophage depletion/rescue	Major-organ H&E; no important abnormalities	Surrogate uptake rather than parent Cur; fewer TAMs may reflect recruitment/depletion as well as reprogramming	D	I	I	D	D	D	D	I
**Liao et al. [45]**	Cur inhibits NF-κB/Bcl-2 and activates caspase/Bax; ICD markers measured	No TAM-specific measurement	Mature DCs 1.77× PBS; CD8+ T cells 1.60× PBS; ICD/cytokine activation	PD1-overexpressing nanovesicle itself provides PD1/PD-L1 blockade; not a conventional separate antibody combination	Integrated ICD + engineered checkpoint-binding carrier	No TAM dependence; no immune-cell depletion test reported	Major-organ H&E and serum chemistry; good biocompatibility	Mechanism is DC/CD8/ICD-centered, not TAM-mediated; DiR tracks carrier rather than Cur	D	I	NR	D	NR	D	D	NR
**Ji et al. [46]**	Tumor-cell EGFR/p-STAT3/PD-L1 reduced; ICD via ROS/mitochondrial damage; no TAM pathway	No TAM measurement	CRT↑; CTL/DC↑; Treg↓; CD8 infiltration in combination 1.9× control	Actual anti-PD-1 monotherapy and nano+laser comparator included	Combo tumor weight 18.81× smaller than anti-PD-1 and 5.52× smaller than nano+laser; not formal synergy calculation	No TAM dependence	Blood/chemistry/organ histology; long-term/systemic toxicity still noted as future need	Multimodal PTT/Cur effects complicate attribution; efficacy dose not explicitly matched to 2.5 mg/kg imaging dose	D	I	NR	D	NR	D	D	NR
**Ding et al. [47]**	PD-L1/CSN5 and ROS-responsive release; ADC IC50 0.5196 vs free Cur 5.504 μg/mL	No TAM measurement	Immune-potentiation claims but nude host limits adaptive checkpoint interpretation	Anti-PD-L1 is covalently integrated in ADC; free anti-PD-L1 + Cur comparator present	ADC superior to free Cur; conventional ICB sensitization not established	No	Major-organ H&E reported	Immunodeficient model; no TAM; antibody-drug conjugate differs from conventional checkpoint combination	I	I	NR	I	NR	I	I	NR
**Xu et al. [48]**	miR-16-5p suppresses PD-L1; Cur/UTMD induces ICD	No TAM measurement	NK/T-cell enrichment and immune activation reported	No checkpoint inhibitor administered	Co-cargo + ultrasound multimodality; cannot attribute all immune effects to Cur	No TAM/immune depletion	Major-organ H&E without obvious lesions	Hepa1-6/BALB/c allogeneic context; no checkpoint	D	I	NR	D	NR	D	NR	NR

								treatment; in vivo Cur mg/kg NR								
**Shiri et al. [50]**	Tumor/spleen STAT4 and IL-12↑; STAT3, IL-10, Arg1↓	Macrophage presence plus tissue-level gene signatures; no cell-resolved functional macrophage assay	No direct lymphocyte/checkpoint functional endpoint	None	Dose-response antitumor effect; no formal multimodal synergy	No	No weight/behavior toxicity reported at studied doses in linked work	Macrophage attribution based mainly on tissue histology/gene expression, not purified TAM function or Cur exposure	I	NR	NR	I	I	NR	NR	NR
**Mukherjee et al. [51]**	Iba1+ microglial ARG1↓/iNOS↑ with directly measured NF-κB/p65 and STAT1 expression/phosphorylation; tumor remission associated with treatment, without demonstration of macrophage-specific causal dependence	Iba1+ microglial phenotype and cell-specific pathway activity measured; tumor remission associated with treatment, but macrophage functional causality not independently tested	Immune-mediated tumor control inferred; no checkpoint therapy	None	Delivery strategies compared; no checkpoint synergy	No macrophage depletion/rescue	No major systemic toxicity signal emphasized	CD68 is also abundant on GL261 tumor cells; targeted adduct therefore not TAM-specific	I	I	I	D	I	I	NR	NR
**Mukherjee et al. [52]**	TAM STAT3/P-STAT3 suppressed; STAT1/P-STAT1↑; ARG1/IL-10↓; iNOS/IL-12↑	Cell-resolved Iba1+ TAM state/function and macrophage recruitment	Activated NK recruitment; CCP effect partially reversed by NK1.1 neutralization; IL-12 blockade reduces NK recruitment	None	Immune mechanism stronger than direct tumor cytotoxicity at sub-IC50 systemic Cur	NK1.1 neutralization + IL-12 blockade; shows NK contribution, not macrophage necessity	Short-course treatment; no comprehensive long-term nanomedicine safety package	NK dependence demonstrated; macrophage necessity not directly tested; TAM parent-Cur unmeasured	I	I	NR	D	D	D	NR	NR
**Mukherjee et al. [53]**	p53/apoptosis + microglial/macrophage state changes in GL261	Cell-resolved Iba1+ TAM/microglia shift: ARG1 decreased and iNOS increased; TrLp produced larger changes than Cur-liposome	Activated NK-cell recruitment within GBM was directly measured; associated with tumor control	None	TriCurin is intrinsically multi-agent; response cannot be assigned to Cur alone	No macrophage depletion/rescue	Preclinical tolerability only	TriCurin is intrinsically multi-agent; Cur-specific attribution is limited despite plasma parent-Cur measurement. No TAM intracellular Cur measurement or macrophage-dependence test.	I	I	NR	I	D	D	NR	NR
**Wu et al. [93]**	Cur+DOX co-delivery increased ROS, CRT exposure, HMGB1 redistribution, and ATP release in 4T1 cells; Cur:DOX 1:1 yielded CI 0.43 in vitro. ER-stress pathway activation/dependence was not directly established.	No TAM measurement	Increased tumor CD8 IHC signal and serum IL-6 (252.73), IFN-γ (1891.72), and GZMB (48.51 pg/mL) in the dual-NP group; supportive immune-activation evidence without direct functional DC/NK/T-cell assays	No checkpoint therapy	Formal in vitro drug synergy; in vivo TIR 84.27 ±1.72% vs 56.38 ±6.61% DOX NP and 70.14 ±3.07% uncoated dual NP	No	Body weight, hemolysis, and organ safety assessments	Multi-agent ICD platform; no TAM/checkpoint mechanism; numeric in-vivo drug doses not reported	D	I	NR	I	NR	I	NR	NR

Evidence coding: D = directly demonstrated within that domain; I = indirect/supportive, partial, or resolution-limited evidence; NR = not measured/reported. Domains are independent rather than cumulative. D1, pharmaceutical/formulation performance; D2, tumor delivery/exposure; D3, TAM-specific uptake plus intracellular parent-Cur exposure; D4, cell-specific pathway modulation; D5, functional TAM reprogramming; D6, downstream immune restoration; D7, checkpoint-therapy response; D8, translational/human relevance. For D2, carrier- or surrogate-dye-based tumor imaging without quantitative measurement of parent Cur in tumor tissue is coded I rather than D, even when the fluorescence signal itself is quantified. For D3, carrier-associated fluorescence or surrogate-dye uptake without quantitative intracellular parent Cur is coded I rather than D. For D4, D denotes directly measured

pathway modulation in the relevant cell. Causal pathway dependence is not inferred from D4 = D and requires a perturbation or rescue experiment; direct molecular target engagement requires independent drug–target binding or occupancy evidence. Marker-only macrophage shifts are generally coded I for D5 unless accompanied by independent functional/cytokine evidence. NR values were not inferred.

Supplementary Table S2. Chemical identity and evidentiary classification of curcumin-related compounds and formulations discussed in the review

Study / ref.	Formulation / system	Chemical identity of Cur-related component	Identity category	Role in study	Evidence class	Reason for classification / interpretive caution
**Dong et al. [40]**	M2pep-Cs-Cur NP	Parent curcumin	Parent Cur; single Cur cargo	TAM-targeted Cur delivery / macrophage remodeling; checkpoint combination	Core	Directly relevant to nanodelivery + TAM axis, but no anti-PD-L1-only arm and no macrophage-dependence test.
**Liu et al. [41]**	Cur-PDA@CM	Parent curcumin	Parent Cur; self-assembled Cur core	Biomimetic tumor delivery, PTT, TAM phenotype modulation	Core	Parent Cur is the drug; macrophage membrane is a carrier/camouflage element and should not be interpreted as TAM targeting.
**Zhang et al. [42]**	Cur-CSNPs	Parent curcumin	Parent Cur	Difunctional photosensitizer and HIF-1α modulator in PDT	Core	Parent Cur is directly loaded; TAM shift is downstream of treated tumor cells and is primarily marker-defined.
**Xiao et al. [43]**	CUR@PPC-aPD-1	Parent curcumin + anti-PD-1	Parent Cur in multi-component therapeutic system	NF-κB inhibition integrated with PD-1 blockade	Core	Cur identity is parent compound; anti-PD-1 is a separate therapeutic component with appropriate single-component comparators.
**Wang et al. [44]**	Cur@CPP-aPD-L1	Parent curcumin + anti-PD-L1	Parent Cur in co-delivery system	NF-κB/TME modulation + checkpoint blockade	Core	Parent Cur is encapsulated and aPD-L1 is surface-linked/released; immune effects arise from both components.
**Liao et al. [45]**	PD1@Cur-PLGA	Parent curcumin	Parent Cur; despite "curcuminoids" in title, experimental cargo is Cur	ICD induction combined with engineered PD1/PD-L1 blockade	Core	Methods explicitly prepare Cur-loaded PLGA. Engineered PD1 nanovesicles independently contribute checkpoint-blocking activity.
**Ji et al. [46]**	Cu9S8@PCM@Cur ± anti-PD-1	Parent curcumin	Parent Cur in thermoresponsive multimodal system	Heat-triggered chemotherapy/ICD and tumor-cell PD-L1 modulation; anti-PD-1 combination	Core	Parent Cur is released from PCM; PTT and anti-PD-1 independently contribute to efficacy.
**Ding et al. [47]**	anti-PD-L1-PBA-Cur ADC	Cur moiety covalently conjugated through ROS-responsive PBA linker	Parent-Cur-derived conjugate / releasable Cur	PD-L1-directed Cur delivery	Core-supportive	Chemically linked Cur is not identical to freely administered parent Cur until linker cleavage; ADC architecture differs from standard ICB combination.
**Xu et al. [48]**	Cur/miR-16-5p nanobubbles	Parent curcumin + miR-16-5p	Parent Cur in multi-agent co-delivery system	Cur-associated ICD + miR-16-mediated PD-L1 suppression	Supportive	No checkpoint inhibitor is administered; PD-L1 suppression cannot be attributed to Cur alone because miR-16-5p is an active co-cargo.
**Shiri et al. [50]**	Dendrosomal curcumin (DNC)	Parent curcumin formulated in dendrosomes	Parent Cur formulation	Macrophage-associated antitumor effects in metastatic breast cancer	Core-supportive	Relevant to macrophage biology, but lacks cell-resolved Cur exposure and modern formulation characterization.
**Mukherjee et al. [51]**	CC-CD68Ab adduct and CCP/Meriva	Curcumin in antibody adduct; commercial phytosomal preparation contains curcuminoids	Parent Cur / curcuminoid formulation	Microglia/macrophage delivery and glioblastoma control	Core-supportive	CD68 is also expressed by GL261 tumor cells; commercial CCP is not a chemically pure parent-Cur-only preparation.
**Mukherjee et al. [52]**	CCP/Meriva phytosomal curcumin	Commercial curcuminoid phytosome	Curcuminoid formulation	TAM/microglia repolarization and NK-dependent tumor control	Core-supportive	Strong macrophage/NK mechanism, but exposure is a phytosomal curcuminoid mixture rather than analytically isolated parent Cur.
**Mukherjee et al. [53]**	Liposomal TriCurin (TrLp)	Curcumin + epicatechin gallate + resveratrol (C:E:R 4:1:12.5)	Multi-agent polyphenol formulation	TAM/microglia repolarization + NK recruitment	Supportive	Cannot assign the observed biological effect specifically to Cur because two active polyphenols are co-formulated at a synergistic ratio.
**Wu et al. [93]**	CMCS-D+C/NPs	Parent curcumin + doxorubicin	Parent Cur in multi-agent chemotherapy system	ICD amplification and immune activation	Supportive	Formal Cur/DOX synergy is shown, but TAM involvement and checkpoint therapy are absent; immune effects are not Cur-specific.
**Zhou et al. [56]**	Tetrahydrocurcumin	Tetrahydrocurcumin	Reduced Cur metabolite/derivative	SPP1/CD44-associated macrophage polarization in colorectal cancer	Supportive	Chemically distinct from parent Cur; useful for curcuminoid biology but should not be cited as direct evidence of parent-Cur pharmacology.
**Zhang et al. [54]**	Hydrazinocurcumin nanoparticles	Hydrazinocurcumin	Synthetic Cur derivative	STAT3-associated TAM re-education	Historical / supportive	Chemically distinct from parent Cur. The 2013 article carries an Expression of Concern [55] and should not serve as a secure evidentiary pillar.

**Guo et al. [100]**	Free Cur + anti-PD-1	Parent curcumin	Parent Cur; non-nano combination	Direct checkpoint-combination evidence in HCC	Supportive	Useful for checkpoint combination logic, but not a nanodelivery or TAM-targeted study.
**Salih et al. [101]**	Free Cur in A549 cells	Parent curcumin	Parent Cur; non-nano	Tumor-cell IFN-γ/STAT1-associated PD-L1 regulation	Supportive	Cell-specific tumor-cell evidence; does not establish macrophage PD-L1 regulation or checkpoint response.
**Zheng et al. [97]**	Free Cur in anaplastic thyroid carcinoma	Parent curcumin	Parent Cur; non-nano	AKT/mTORC1/STAT3/PD-L1 modulation and CD8-related antitumor immunity	Supportive	Supports tumor-cell PD-L1/immune mechanism but is not evidence of TAM-targeted nanodelivery.

Table S2 note: "Parent Cur" denotes curcumin itself; "curcuminoid formulation" denotes a preparation that is not analytically equivalent to isolated parent Cur; "derivative/metabolite" denotes a chemically distinct Cur-related molecule; "multi-agent" denotes systems containing another active therapeutic cargo or polyphenol whose contribution cannot be assigned to Cur without component controls. "Core" identifies studies directly informing the review's nanodelivery–TAM–checkpoint axis; "supportive" identifies studies that inform only part of that axis or use chemically distinct/multi-agent preparations. Reference [54] should be interpreted together with the Expression of Concern cited as [55] in the revised manuscript.

Abbreviations: ADC, antibody-drug conjugate; BMDM, bone-marrow-derived macrophage; CM, cell membrane; CPP, CDM-PEG-PDPA micelle platform; Cur, curcumin; DC, dendritic cell; DLE/DLC, drug loading efficiency/capacity; DL, drug loading; DOX, doxorubicin; EE, encapsulation efficiency; HCC, hepatocellular carcinoma; ICD, immunogenic cell death; ICB, immune-checkpoint blockade; IHC, immunohistochemistry; IP, intraperitoneal; IV, intravenous; LC-MS, liquid chromatography-mass spectrometry; NIR, near-infrared; NR, not measured/reported; NV, nanovesicle; PDI, polydispersity index; PDT, photodynamic therapy; PLGA, poly(lactic-co-glycolic acid); PTT, photothermal therapy; TAM, tumor-associated macrophage; TIR, tumor inhibition rate; TME, tumor microenvironment; TNBC, triple-negative breast cancer; US, ultrasound; UTMD, ultrasound-targeted microbubble destruction.

References Cited in the Supplementary Information (Numbering Corresponds to the Main Manuscript)

Note: Reference numbers used throughout this Supplementary Information correspond to the numbering in the main manuscript and are not independently renumbered.

[40]. Dong, X.; Wang, X.; Zheng, X.; Jiang, H.; Liu, L.; Ma, N.; Wang, S. Targeted Nanoparticle Delivery System for Tumor-Associated Macrophage Reprogramming to Enhance TNBC Therapy. Cell Biology and Toxicology 2025, 41, 58. https://doi.org/10.1007/s10565-025-10001-1.

[41]. Liu, X.; Miao, X.; Bo, S.; Deng, X.; Zhang, Z.; Zheng, Y. Macrophage Membrane-Coated Self-Assembled Curcumin Nanoparticle Missile for the Treatment of Colorectal Cancer. Journal of Drug Delivery Science and Technology 2024, 91, 105237. https://doi.org/10.1016/j.jddst.2023.105237.

[42]. Zhang, L.-J.; Huang, R.; Shen, Y.-W.; Liu, J.; Wu, Y.; Jin, J.-M.; Zhang, H.; Sun, Y.; Chen, H.-Z.; Luan, X. Enhanced Anti-Tumor Efficacy by Inhibiting HIF-1α to Reprogram TAMs via Core-Satellite Upconverting Nanoparticles with Curcumin Mediated Photodynamic Therapy. Biomaterials Science 2021, 9, 6403–6415. https://doi.org/10.1039/D1BM00675D.

[43]. Xiao, Z.; Su, Z.; Han, S.; Huang, J.; Lin, L.; Shuai, X. Dual pH-Sensitive Nanodrug Blocks PD-1 Immune Checkpoint and Uses T Cells to Deliver NF-κB Inhibitor for Antitumor Immunotherapy. Science Advances 2020, 6, eaay7785. https://doi.org/10.1126/sciadv.aay7785.

[44]. Wang, T.; Lin, M.; Mao, J.; Tian, L.; Gan, H.; Hu, X.; Yan, L.; Long, H.; Cai, J.; Zheng, X.; et al. Inflammation-Regulated Nanodrug Sensitizes Hepatocellular Carcinoma to Checkpoint Blockade Therapy by Reprogramming the Tumor Microenvironment. ACS Applied Materials & Interfaces 2022, 14, 49542–49554. https://doi.org/10.1021/acsami.2c14448.

[45]. Liao, Y.; Zhao, C.; Pan, Y.; Guo, Y.; Liu, L.; Wu, J.; Zhang, Y.; Rao, L.; Li, Q. Genetically Engineered Cellular Nanoparticles Loaded with Curcuminoids for Cancer Immunotherapy. Theranostics 2024, 14, 6409–6425. https://doi.org/10.7150/thno.99033.

[46]. Ji, A.; Yang, P.; Ma, B.; Li, J.; Yu, Y.; Zhang, X.; Chen, P.; Yao, H.; Shi, L.; Hu, H.; et al. Photothermal-Responsive Curcumin-Loaded Copper-Based Nanocomposites for Targeted Drug Release and Combined Immunotherapy. Journal of Colloid and Interface Science 2025, 698, 138042. https://doi.org/10.1016/j.jcis.2025.138042.

[47]. Ding, J.; Liu, Z.; Liu, S.; Xie, X.; Yin, Q.; Lu, W.; Wang, W.; Zhang, Y. Preparation and Anti-Tumor Ability Evaluation of Anti-PD-L1 Conjugated Curcumin in Colon Cancer. International Journal of Biological Macromolecules 2025, 306, 141563. https://doi.org/10.1016/j.ijbiomac.2025.141563.

[48]. Xu, Q.; Yang, S.; Han, J.; Liu, C.; Zhao, Y.; Liu, Y. Ultrasound-Responsive Curcumin/MiR-16-5p Co-Loaded Nanobubbles Enhance Antitumor Immunity in Hepatocellular Carcinoma via ICD Induction and PD-L1 Suppression. Toxicology and Applied Pharmacology 2026, 515, 117966. https://doi.org/10.1016/j.taap.2026.117966.

[50]. Shiri, S.; Alizadeh, A.M.; Baradaran, B.; Farhangi, B.; Shanehbandi, D.; Khodayari, S.; Khodayari, H.; Tavassoli, A. Dendrosomal Curcumin Suppresses Metastatic Breast Cancer in Mice by Changing M1/M2 Macrophage Balance in the Tumor Microenvironment. Asian Pacific Journal of Cancer Prevention 2015, 16, 3917–3922. https://doi.org/10.7314/APJCP.2015.16.9.3917.

[51]. Mukherjee, S.; Baidoo, J.; Fried, A.; Atwi, D.; Dolai, S.; Boockvar, J.; Symons, M.; Ruggieri, R.; Raja, K.; Banerjee, P. Curcumin Changes the Polarity of Tumor-Associated Microglia and Eliminates Glioblastoma. International Journal of Cancer 2016, 139, 2838–2849. https://doi.org/10.1002/ijc.30398.

[52]. Mukherjee, S.; Fried, A.; Hussaini, R.; White, R.; Baidoo, J.; Yalamanchi, S.; Banerjee, P. Phytosomal Curcumin Causes Natural Killer Cell-Dependent Repolarization of Glioblastoma (GBM) Tumor-Associated Microglia/Macrophages and Elimination of GBM and GBM Stem Cells. Journal of Experimental & Clinical Cancer Research 2018, 37, 168. https://doi.org/10.1186/s13046-018-0792-5.

[53]. Mukherjee, S.; Baidoo, J.N.E.; Sampat, S.; Mancuso, A.; David, L.; Cohen, L.S.; Zhou, S.; Banerjee, P. Liposomal TriCurin, a Synergistic Combination of Curcumin, Epicatechin Gallate and Resveratrol, Repolarizes Tumor-Associated Microglia/Macrophages, and Eliminates Glioblastoma (GBM) and GBM Stem Cells. Molecules 2018, 23, 201. https://doi.org/10.3390/molecules23010201.

[54]. Zhang, X.; Tian, W.; Cai, X.; Wang, X.; Dang, W.; Tang, H.; Cao, H.; Wang, L.; Chen, T. Hydrazinocurcumin Encapsuled Nanoparticles "Re-Educate" Tumor-Associated Macrophages and Exhibit Anti-Tumor Effects on Breast Cancer Following STAT3 Suppression. PLOS ONE 2013, 8, e65896. https://doi.org/10.1371/journal.pone.0065896.

[55]. The PLOS ONE Editors. Expression of Concern: Hydrazinocurcumin Encapsuled Nanoparticles "Re-Educate" Tumor-Associated Macrophages and Exhibit Anti-Tumor Effects on Breast Cancer Following STAT3 Suppression. PLOS ONE 2019, 14, e0220672. https://doi.org/10.1371/journal.pone.0220672.

[56]. Zhou, M.; Li, R.; Lian, G.; Yang, M.; Li, L.; Yin, Z.; Li, G.; Zhao, J.; Tan, R. Tetrahydrocurcumin Alleviates Colorectal Tumorigenesis by Modulating the SPP1/CD44 Axis and Preventing M2 Tumor-Associated Macrophage Polarization. Phytomedicine 2025, 141, 156674. https://doi.org/10.1016/j.phymed.2025.156674.

[93]. Wu, Y.; Gao, J.; Lu, Y.; Zhao, Y.; Wan, X.; Zhang, H.; Cai, X. Studies on the Anti-Tumor Effects of Curcumin Synergizing with Doxorubicin in Inducing Immunogenic Cell Death. Nanomedicine: Nanotechnology, Biology and Medicine 2026, 74, 102951. https://doi.org/10.1016/j.nano.2026.102951.

[97]. Zheng, J.; Liu, W.; Wang, X.; Li, H.; Wang, Z.; Ai, Z. Curcumin Enhances Anti-Tumor Immunity in Anaplastic Thyroid Carcinoma by Elevating CD8+ T Cell Function and Downregulating the AKT/mTORC1/STAT3/PD-L1 Axis. Pathology - Research and Practice 2025, 269, 155898. https://doi.org/10.1016/j.prp.2025.155898.

[100]. Guo, L.; Li, H.; Fan, T.; Ma, Y.; Wang, L. Synergistic Efficacy of Curcumin and Anti-Programmed Cell Death-1 in Hepatocellular Carcinoma. Life Sciences 2021, 279, 119359. https://doi.org/10.1016/j.lfs.2021.119359.

[101]. Salih, D.J.; Barsoom, S.H.; Ahmed, G.F.; Hussien, S.Q.; Al Ismaeel, Q.; Alasady, A.A.B.; Alsalim, T.A.; Salih, A.M. Curcumin Inhibits IFN-γ Induced PD-L1 Expression via Reduction of STAT1 Phosphorylation in A549 Non-Small Cell Lung Cancer Cells. Saudi Pharmaceutical Journal 2025, 33, 16. https://doi.org/10.1007/s44446-025-00018-2.
